# Apolipoprotein E4 Effects a Distinct Transcriptomic Profile and Dendritic Arbor Characteristics in Hippocampal Neurons Cultured *in vitro*

**DOI:** 10.3389/fnagi.2022.845291

**Published:** 2022-04-29

**Authors:** Jenny R. Diaz, Mitchell Martá-Ariza, Alireza Khodadadi-Jamayran, Adriana Heguy, Aristotelis Tsirigos, Joanna E. Pankiewicz, Patrick M. Sullivan, Martin J. Sadowski

**Affiliations:** ^1^Department of Neurology, New York University Grossman School of Medicine, New York, NY, United States; ^2^Department of Pathology, New York University Grossman School of Medicine, New York, NY, United States; ^3^Department of Biochemistry and Pharmacology, New York University Grossman School of Medicine, New York, NY, United States; ^4^Department of Medicine (Geriatrics), Duke University School of Medicine, Durham, NC, United States; ^5^Durham VA Medical Center’s, Geriatric Research Education and Clinical Center, Durham, NC, United States; ^6^Department of Psychiatry, New York University Grossman School of Medicine, New York, NY, United States

**Keywords:** Alzheimer’s disease, ApoE, gene ontology analysis, KEGG enrichment analysis, neurodegeneration, primary hippocampal cell cultures, synaptic plasticity, transcriptome

## Abstract

The *APOE* gene is diversified by three alleles *ε2*, *ε3*, and *ε4* encoding corresponding apolipoprotein (apo) E isoforms. Possession of the *ε4* allele is signified by increased risks of age-related cognitive decline, Alzheimer’s disease (AD), and the rate of AD dementia progression. ApoE is secreted by astrocytes as high-density lipoprotein-like particles and these are internalized by neurons upon binding to neuron-expressed apoE receptors. ApoE isoforms differentially engage neuronal plasticity through poorly understood mechanisms. We examined here the effects of native apoE lipoproteins produced by immortalized astrocytes homozygous for *ε2*, *ε3*, and *ε4* alleles on the maturation and the transcriptomic profile of primary hippocampal neurons. Control neurons were grown in the presence of conditioned media from *Apoe*^–/–^ astrocytes. ApoE2 and apoE3 significantly increase the dendritic arbor branching, the combined neurite length, and the total arbor surface of the hippocampal neurons, while apoE4 fails to produce similar effects and even significantly reduces the combined neurite length compared to the control. ApoE lipoproteins show no systemic effect on dendritic spine density, yet apoE2 and apoE3 increase the mature spines fraction, while apoE4 increases the immature spine fraction. This is associated with opposing effects of apoE2 or apoE3 and apoE4 on the expression of NR1 NMDA receptor subunit and PSD95. There are 1,062 genes differentially expressed across neurons cultured in the presence of apoE lipoproteins compared to the control. KEGG enrichment and gene ontology analyses show apoE2 and apoE3 commonly activate expression of genes involved in neurite branching, and synaptic signaling. In contrast, apoE4 cultured neurons show upregulation of genes related to the glycolipid metabolism, which are involved in dendritic spine turnover, and those which are usually silent in neurons and are related to cell cycle and DNA repair. In conclusion, our work reveals that lipoprotein particles comprised of various apoE isoforms differentially regulate various neuronal arbor characteristics through interaction with neuronal transcriptome. ApoE4 produces a functionally distinct transcriptomic profile, which is associated with attenuated neuronal development. Differential regulation of neuronal transcriptome by apoE isoforms is a newly identified biological mechanism, which has both implication in the development and aging of the CNS.

## Introduction

Apolipoprotein (apo) E is a 34-kDa protein, which regulates lipid metabolism, influences synaptic plasticity, and controls risk of sporadic Alzheimer’s disease (AD) (Yamazaki et al., 2019). Under physiological conditions, apoE is secreted by astrocytes in the form of high-density lipoprotein (HDL) like particles (Gong et al., 2002; Morikawa et al., 2005; Xu et al., 2006; Holtzman et al., 2012). Astrocyte expressed ATP-binding cassette transporter A1 is responsible for the apoE lipidation (Wahrle et al., 2005, 2008). ApoE lipoproteins are internalized by neurons upon binding to several types of receptors (Holtzman et al., 2012; Yamazaki et al., 2019). Within the family of CNS expressed apoE receptors the low-density lipoprotein receptor (LDLR) has been found foremost critical in controlling brain apoE level (Fryer et al., 2005; Kim et al., 2009; Castellano et al., 2012). The *APOE* gene is located in the human chromosome 19 and is diversified by three alleles *ε2*, *ε3*, and *ε4* (Weisgraber et al., 1981) with uneven prevalence in the population ε*3* > ε*4* > ε*2* (Ordovas et al., 1987; Yamazaki et al., 2019). Inheritance of the *ε4* allele increases risk of sporadic AD, which is elevated ∼3 and ∼15-fold in ε*4* hetero and homozygotes compared to ε*3/*ε*3* homozygotes, representing ∼50% of the population, respectively (Corder et al., 1993). The least common ε*2* allele reduces the AD risk (Corder et al., 1994; Reiman et al., 2020). Besides having elevated odds ratio for sporadic AD, ε*4* carriers also remain at higher risk for age-related cognitive decline (Greenwood et al., 2000; Caselli et al., 2007, 2009, 2011; Luck et al., 2015), worse outcome of the traumatic brain injury (Collie et al., 2004; Ariza et al., 2006; Merritt et al., 2018), and more aggressive clinical course of dementia once they develop symptoms of AD (Martins et al., 2005; Cosentino et al., 2008; Shi et al., 2017; Chen X. R. et al., 2021). These multiple biological effects differentially regulated by the *APOE* genotype are driven by structural differences across apoE isoforms, which are encoded by respective *APOE* alleles. The three apoE isoforms differ in the presence of cysteine and arginine at the residues 112 and 158: apoE2 (cys/cys), apoE3 (cys/arg), and apoE4 (arg/arg) (Sadowski and Wisniewski, 2006). These minute differences in the amino acidic sequence have impactful functional significance resulting in reduced receptor binding affinity of apoE2 and increased intramolecular domain interaction and reduced lipid content of apoE4 (Weisgraber et al., 1981; Frieden et al., 2017; Chen Y. et al., 2021). Phospholipids and cholesterol ferried by apoE lipoproteins play a crucial metabolic role in maintaining synaptic terminals (Mauch et al., 2001). Several studies have suggested that besides lipid delivery apoE impacts neurite outgrowth, synapse formation, and synaptic ion channel function, which all are involved in neuronal network plasticity and maintenance, and that these effects are differentially regulated by various apoE isoforms (Qiu et al., 2004; Lane-Donovan et al., 2016). Paradoxically, despite being a risk factor for age related cognitive decline, the ε*4* allele was found to be associated with enhanced performance on some cognitive tasks at younger age (Yu et al., 2000; Mondadori et al., 2007; Jochemsen et al., 2012; Rusted et al., 2013). This oppositional effect of the ε*4* allele, being an expedient characteristic in young individuals while becoming a disadvantageous one during the aging is known as the antagonistic pleiotropy (Tuminello and Han, 2011; Rusted and Carare, 2015; Di Battista et al., 2016). Despite the recognized role apoE plays in neuronal network maintenance and plasticity, mechanisms through which apoE isoforms differentially engages synaptic biology remain obscure. To this end we analyzed differences in dendritic arbor characteristics and transcriptomic profile across primary hippocampal neurons, which were cultured in the presence of native apoE lipoproteins between 10 and 17 day *in vitro* (DIV). Compared to neurons grown in the presence of apoE2 and apoE3, those grown in the presence of apoE4 feature lesser complexity of dendritic arbor and increased proportion of immature dendritic spine types. Transcriptomic analysis shows that apoE4 engages a distinctly different set of genes and signaling pathways than those engaged by apoE2 and apoE3. Differential regulation of neuronal transcriptome by apoE isoforms revealed by our work is a newly identified biological mechanism, with possible implication both in the development and aging of the neuronal networks.

## Materials and Methods

### Materials

Immortalized astrocytic lines, which produce natively lipidated apoE lipoproteins were originally derived from the human *APOE* targeted replacement mice (Sullivan et al., 1997, 1998; Knouff et al., 1999). These lines are homozygous for ε*2*, ε*3*, or ε*4* human *APOE* alleles. Development of immortalized astrocytic lines and characterization of apoE lipoproteins they produce have been described previously (Morikawa et al., 2005). Immortalized *Apoe^–/–^* astrocytes derived from the *Apoe^–/–^* mice (Zhang et al., 1992) were used to produce control conditioned media lacking human apoE lipoproteins. Cell culture media, supplements, and reagents used for isolation and culture of astrocytes and neurons were of Gibco™ brand and were purchased from Thermo Fisher Scientific (Waltham, MA, United States). Proteomic reagents were of Pierce™ brand and they also were purchased from Thermo Fisher Scientific. Kits and reagents for RNA extractions were obtained from Qiagen Sciences Inc. (Germantown, MD, United States). Recombinant human apoE was bought from Leinco Technologies (St. Louis, MO, United States). Primary and secondary antibodies were from different sources individually indicated in the text. All other chemicals and reagents were obtained either from Sigma-Aldrich (St. Louis, MO, United States) or from Thermo Fisher Scientific unless indicated otherwise.

### Culturing of Immortalized Astrocytes

**FIGURE 1:**
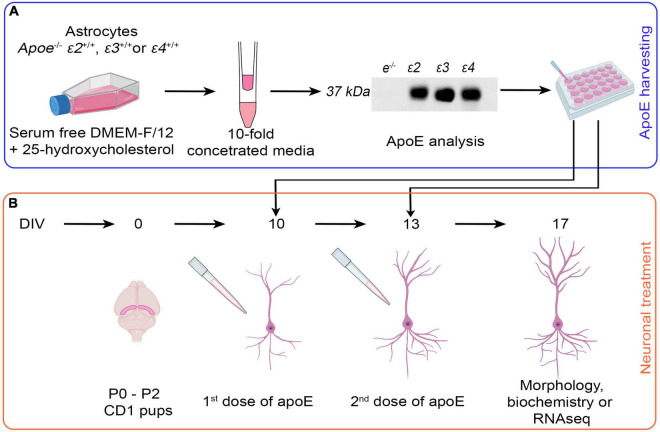
Schema of the experimental design. **(A)** Harvesting of native apoE lipoproteins. Immortalized *Apoe*^–/–^ astrocytes, and astrocytes homozygous for human *APOE* ε*2*, ε*3*, and ε*4* alleles are grown in the serum free DMEM/F-12 medium supplemented by 25-hydroxycholesterol for 48 h. Conditioned media are collected and 10-fold concentrated using 10 kDa MWCO Amicon Centrifugal Filter Units. ApoE level is verified by ELISA and balanced out by adding 10-fold concentrated media from *Apoe*^–/–^ astrocytes if needed. ApoE lipoprotein preparations or control media (10-fold concentrated *Apoe*^–/–^ conditioned media) are added to cultured neurons. **(B)** Treatment of primary hippocampal neurons with apoE lipoproteins. Primary hippocampal neuronal cultures are established from P0–P2 pups of CD1 mice in aseptic conditions. At 10 DIV and again at 13 DIV the control media or apoE lipoproteins are added. At 17 DIV the experiment is concluded and the neurons are subjected to morphological, biochemical, or transcriptomic analyses. The gradual development of dendritic arbor during *in vitro* maturation is schematically depicted in **(B)**. The diagram was created with the aid of BioRender.com.

Immortalized *Apoe^–/–^* astrocytes and those homozygous for ε*2*, ε*3*, and ε*4* human *APOE* alleles were maintained in the Dulbecco’s Modified Eagle Medium/Nutrient Mixture F-12 (DMEM/F-12) supplemented with 10% heat-inactivated fetal bovine serum (FBS), 1 mM sodium pyruvate, and 0.4% geneticin. To harvest the apoE lipoproteins, the astrocytes were plated in T-25 flasks and grown until they reach 75–85% confluence. Then the standard growth medium was replaced with serum free DMEM/F-12 supplemented with 1× N-2 supplement and 3 mM 25-hydroxycholesterol to enhance apoE production. The astrocytes were grown in the serum free DMEM/F-12 for 48 h. prior to harvesting the conditioned media containing apoE lipoproteins (Figure 1A).

### Concentration of ApoE Lipoproteins

**FIGURE 2:**
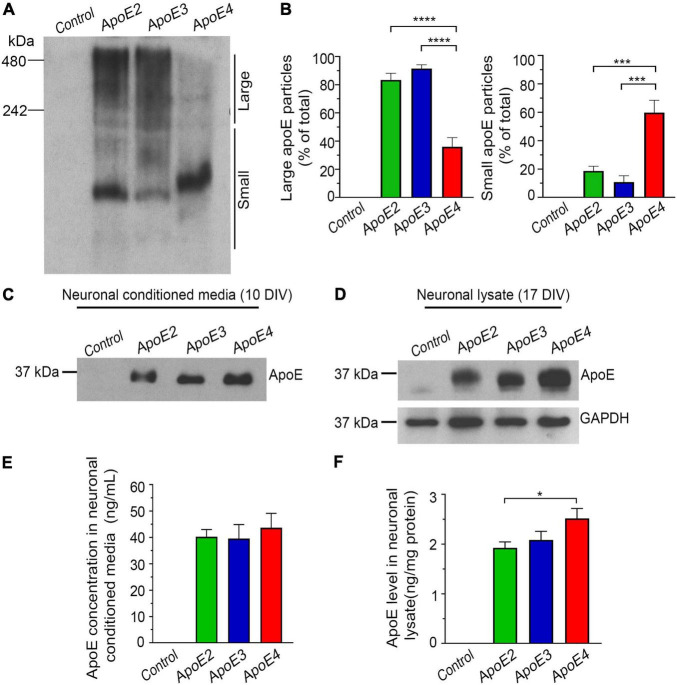
Characterization of the native apoE lipoprotein particles and their uptake by hippocampal neurons *in vitro*. Shown are Western blot analysis **(A)** and densitometric quantification **(B)** of apoE lipoproteins from astrocytic conditioned media, which are resolved under native conditions. The control denotes conditioned media from *Apoe^–/–^* astrocytes. The left and the right panel in **(B)** represent densitometric quantification of apoE protein band optical densities above and below the 242 kDa molecular weight marker, which is arbitrary used to discern large and small apoE lipoprotein particles, respectively. Whilst apoE2 and apoE3 signal dominates above the 242 kDa marker the apoE4 signal is localized mainly below it, what indicates reduced lipidation of apoE4 compared to apoE2 and apoE3. Shown is Western blot analysis of the apoE protein from the conditioned media of primary hippocampal neurons **(C)** and from the neuronal lysate **(D)**, which are resolved under reducing conditions. Neurons were treated with the control media (from *Apoe^–/–^* astrocytes) or indicated apoE lipoproteins. The conditioned media were sampled approximately 20 min. after adding the first dose of apoE lipoproteins, while neuronal lysates are from 17 DIV neurons collected at the conclusion of the experiment. Included also is Western blot analysis of GAPDH, used as the loading control for the Western blotting analysis of neuronal lysate. Shown are ELISA measures of apoE level in the neuronal media **(E)** and in the neuronal lysates **(F)**, which were sampled at the beginning and at the conclusion of the experiment, respectively. Consistently with the Western analysis, the ELISA measurements show comparable level of all three apoE isoforms in the neuronal media at the beginning of the experiment and elevated level of intraneuronal apoE4 compared to apoE2 and apoE3 at the conclusion of the experiment. Values in **(B)**, represent mean and SEM from five independent experiments, while those in **(E,F)** represent mean and SEM from eight independent experiments. **(B,E,F)** *p* < 0.0001 (ANOVA); *p < 0.05, ***p < 0.001, and ****p < 0.0001 (Holm’s–Sidak’s *post hoc* test).

Neat astrocytic media were 10-fold concentrated to minimize the amount of DMEM/F-12 introduced to the primary neuronal cultures. This was done by centrifugation of the media in Amicon^®^ Ultra-15 Centrifugal Filter Units with 10 kDa molecular weight cut off (MWCO) cellulose membranes (MilliporeSigma, Burlington, MA, United States) at 4,000 × *g* and 4°C (Figure 1A). To limit precipitation of apoE lipoproteins on the filter membrane the Amicon Filter Units were primed with 0.1 mg/ml solution of bovine serum albumin (BSA) in 0.01 M phosphate buffered saline (PBS) (Pankiewicz et al., 2021). The apoE level in 10-fold concentrated media was quantified using enzyme-linked immunosorbent assay (ELISA) (Pankiewicz et al., 2014) and the differences between batches were balanced out by adding 10-fold concentrated media from *Apoe^–/–^* astrocytes (Figure 1A). Lipidation status of the apoE lipoprotein particles was examined by Tris-Glycine native gel electrophoresis followed by Western blotting (Figures 2A,B).

### Cultures of Primary Hippocampal Neurons and Their Treatment With ApoE Lipoproteins

Dissociated cultures of primary hippocampal neurons were established from P0–P2 old pups of CD1 mice. Following rapid decapitation, the brains were extracted from the skulls and the hippocampi were dissected out in aseptic conditions (Figure 1B). Cell suspension was prepared by digesting the tissue with 30 units of Papain (Worthington Biochemical Corporation, Lakewood, NJ, United States) and 200 units of DNAse I (Worthington Biochemical Corporation) for 30 min at 37°C, triturating with fire-polished glass pipettes, and filtering through a 40 μm cell strainer (Kuszczyk et al., 2013). The neurons were counted and plated into 6-well plates in the amount of 50,000 per well for cytochemical analysis or in the amount of 500,000 per well for biochemical or RNA sequencing analyses. For the former type of analysis the neurons were seeded on poly-D-lysine coated, round, 18 mm in diameter coverslips (Ted Pella, Inc., Redding, CA, United States) placed on the bottom of the well. For the latter type of analysis, the neurons were seeded directly on the poly-D-lysine coated well bottom. Primary hippocampal neurons were cultured in the Neurobasal medium supplemented with 2% B-27 supplement, 2 mmol/L glutamine, 50 mg/mL of streptomycin, and 100 U/mL penicillin. One-third of the medium volume was replaced every third day. On the third DIV, primary hippocampal neurons were treated with 5 μmol/L cytosine-β-D-arabinoside to prevent growth of non-neuronal cells. On the 10 DIV and again on the 13 DIV apoE-enriched, 10-fold concentrated astrocytic conditioned media were added to the Neurobasal medium (1:10 vol/vol). The experiment was concluded at 17 DIV when the primary neuronal cultures were fixed for cytochemical characterization or lysed for biochemical or transcriptomic analyses. The concentration of apoE in the neuronal conditioned media at the commencement of the experiment and the amount of apoE internalized by neurons at the conclusion of the experiment were quantified by ELISA and by polyacrylamide gel electrophoresis (PAGE) performed under reducing conditions, followed by Western blotting (Figures 2C–F). The control neurons were treated on 10 and 13 DIV with 10-fold concentrated conditioned media from immortalized *Apoe^–/–^* astrocytes (1:10 vol/vol). The absence of apoE in the conditioned media from *Apoe^–/–^* astrocytes was confirmed using apoE ELISA and Western blotting. Likewise, the absence of apoE in the conditioned media from the control neurons at the commencement of the experiment and in their lysates at the conclusion of the experiment was confirmed (Figure 2). Contamination of dissociated neuronal cultures by native astrocytes was monitored by immunostaining against glial fibrillary acidic protein (GFAP) using a rabbit polyclonal anti-GFAP antibody (1:2000; Dako/Agilent Technologies; Santa Clara, CA, United States) and Alexa Fluor 488 conjugated goat anti-rabbit IgG secondary antibody (1:500; Jackson ImmunoResearch Laboratories Inc.; West Grove, PA, United States) followed by a nuclear counterstaining with 4′,6-diamidino-2-phenylindole (DAPI) (Pankiewicz et al., 2020). The number GFAP-labeled astrocytes was then manually enumerated in test fields 1.067 mm × 1.334 mm. Ten randomly selected test fields per coverslip from three independently established cultures per treatment condition were analyzed at 17 DIV. The degree of native astrocytic contamination ranged around 5.5% of all cells in the culture and did not vary significantly across treatment conditions (Supplementary Figure 1).

### ApoE Enzyme-Linked Immunosorbent Assay

Conditioned media from astrocytic or neuronal cultures were first treated with 0.5% sodium deoxycholate for 45 min at 37°C to delipidate apoE. Lysates of primary hippocampal neuronal cultures were prepared as previously described (Kuszczyk et al., 2013) and centrifuged at 10,000 × *g* for 20 min at 4°C to remove cellular debris. ApoE level was determined by sandwich ELISA utilizing HJ15.7 mouse monoclonal antibody (mAb) anti human apoE (Shi et al., 2017) as the capture antibody (0.1 μg/mL) (gift of D. M. Holtzman) and biotinylated goat anti-human apoE polyclonal antibody (1:5,000) (Meridian Life Science, Inc., Memphis, TN, United States) as the detection antibody (Pankiewicz et al., 2014). The color reaction was obtained using streptavidin conjugated horseradish peroxidase (HRP) (Fitzgerald Industries, Acton, MA, United States) and 3, 3′, 5, 5′- tetramethylbenzidine Peroxidase EIA Substrate Kit (Bio-Rad Laboratories, Inc., Hercules, CA, United States). Colorimetric ELISA readouts were converted to the actual apoE concentrations using standard curves prepared from increasing dilutions of recombinant human apoE. The level of apoE in the conditioned media was expressed in ng of apoE protein per mL while that in the cell lysate in ng of apoE protein per mg of the total protein, which was assessed by the Pierce BCA Protein Assay Kit following manufacturer provided instruction.

### ApoE Western Blotting

Western blots of gels run under native conditions were used to characterize apoE lipoproteins secreted by astrocytes to the conditioned media. Samples of 10-fold concentrated media were mixed with equal amounts of sample buffer containing Tris HCl 50 mM pH 6.8, 10% Glycerol, and 0.004% Coomassie blue G250 and immediately loaded on the pre-casted 4–20% Novex Tris-Glycine native gel and electrophoresed over 4 h at room temperature using Tris-Glycine Native Running Buffer. Following the electrophoresis, native gels were incubated with 1% SDS and 50 mM Tris pH 6.8 for 30 min, and electroblotted into nitrocellulose membranes over 3 h. The membranes were blocked with 5% soy milk diluted in the SuperBlock blocking buffer and incubated overnight at 4°C with polyclonal goat anti-human apoE antibody (1:7,000; Meridian Life Science, Inc.). ApoE-antibody complexes were visualized using HRP-linked donkey anti-goat IgG antibody (1:30,000; Santa Cruz Biotechnology Inc., Dallas, TX, United States) followed by SuperSignal West Pico PLUS Chemiluminescent Substrate (Thermo Fisher Scientific). Membrane autoradiographs were then obtained and digitized as previously described (Asuni et al., 2013, 2014; Pankiewicz et al., 2019). The resulting images were densitometrically analyzed using NIH ImageJ2 (FIJI) (Bethesda, MD, United States) (Schindelin et al., 2012).

Western blots of gels run under reducing conditions were used to characterize apoE levels in the neuronal conditioned media and neuronal lysates. Samples of each were mixed with equal amounts of tricine sample buffer and beta-mercaptoethanol, boiled at 95°C for 5 min., resolved using 12.5% SDS-PAGE protein gels, and transferred into nitrocellulose membranes. Detection and quantification of apoE in the nitrocellulose membranes was performed analogously to that described for Western blots run under native conditions.

### Quantitative Analysis of Neuronal Dendritic Arbor

**FIGURE 3:**
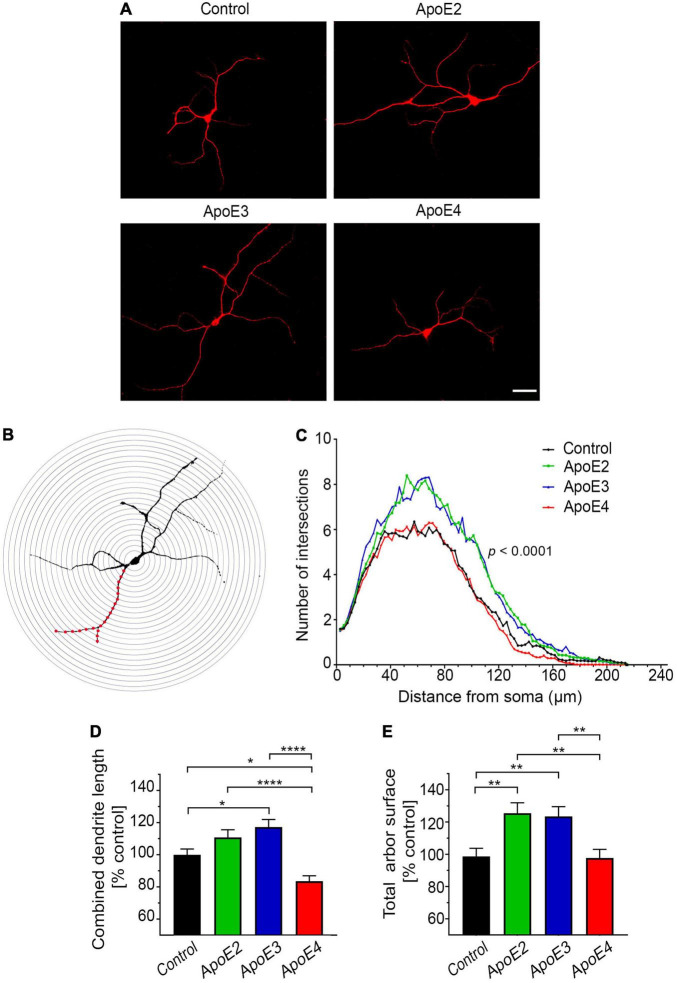
ApoE isoforms differentially regulate development of the dendritic arbor. **(A)** Representative microphotographs of 17 DIV hippocampal neurons grown in the presence of control media (from *Apoe^–/–^* astrocytes) or indicated apoE lipoproteins, which were stained against MAP-2. **(B)** Shown is an example of Sholl’s grid (a set of concentric circles 3 μm apart) overlying MAP-2 immunostained neuron. **(C)** Sholl’s analysis of 17 DIV primary hippocampal neurons, which were treated with the control media or indicated apoE lipoproteins. The radius of concentric circles from the center of the neuron is indicated on the abscissa, while the number of intersections made between the circle of a given radius and the dendritic arbor is given on the ordinate. Values represent mean number of intersections from 54 neurons per group (up to 20 neurons per culture from three independent cultures). SEM are omitted for presentation clarity. Also shown are analyses of the total arbor length **(D)** and the total arbor surface **(E)** of 17 DIV neurons treated with control media or indicated apoE lipoproteins. Values are expressed relative to those in neurons treated with control media and represent mean + SEM from 55 neurons per treatment (up to 20 neurons from each of three independent cultures). ApoE2 or apoE3 treatment significantly increases the number of dendritic branches and the total arbor surface of primary hippocampal neurons, while apoE4 has no significant effect on the number of dendritic branches and the total arbor surface compared to the control neurons, while it significantly reduces the combined dendritic length. **(B)** *p* < 0.0001 (two-way ANOVA); *p* < 0.0001 denotes *post hoc* significance (Holm’s–Sidak’s test) between apoE2 or apoE3 treated neurons and those apoE4 treated or control neurons. *Post hoc* difference between apoE2 and apoE3 treated neurons is not significant and it is not shown on the graph. *Post hoc* difference between the control and apoE4 treated neurons is *p* < 0.05, also is not shown on the graph. **(D,E)** *p* < 0.001 (ANOVA); *p < 0.05, **p < 0.01, and ****p < 0.0001 (Holm’s–Sidak’s *post hoc* test). Scale bar 40 μm in **(A)**.

Primary hippocampal neurons on the cover slips were thrice washed with PBS, fixed with 4% paraformaldehyde (PFA) in 0.1 M phosphate buffer (PB) pH 7.4 over 15 min., thrice washed with PBS again, and permeabilized with 0.25% Triton X-100 in PBS. Then they were washed with PBS containing 0.05% Tween-20 and sequentially blocked with 10% BSA, and avidin/biotin kit (Vector Laboratories; Burlingame, CA, United States). The neuronal cytoskeleton was immunostained using a mouse mAb against microtubule-associated protein 2 (MAP2) (1:500; Sigma-Aldrich) followed by anti-mouse IgG biotinylated secondary antibody (1:500; Vector Laboratories) followed by Cyanine 3 (Cy3) conjugated streptavidin (1:1,000). Immunostained neurons were photographed under 40× objective magnification using high-sensitivity, cooled, monochrome DS-Qi1Mc camera attached to 80i Nikon fluorescent microscope (Nikon Corp., Tokyo, Japan) (Kuszczyk et al., 2013). Digitized neuronal images were analyzed using NIH ImageJ2 (FIJI) (Bethesda, MD, United States) (Schindelin et al., 2012). The dendritic arbor was traced using the simple neurite tracer plugin for NIH ImageJ2 (Longair et al., 2011) and the lengths of all dendritic branches were summed to obtain the combined dendritic length of a neuron. In addition, we quantified the arbor surface area, created by joining the tips of the most far out reaching dendrites using the Image J freehand tool (Wang et al., 2019). Dendrite branching was examined by the Sholl’s analysis (Binley et al., 2014), which enumerates the cross points between dendritic branches and the set of concentric circles spaced 3 μm apart from the center of the neuronal perikaryon (Figure 3B). Twenty randomly selected neurons per culture from three independent cultures per treatment condition were analyzed for all three parameters.

### Dendritic Spine Analysis

**FIGURE 4:**
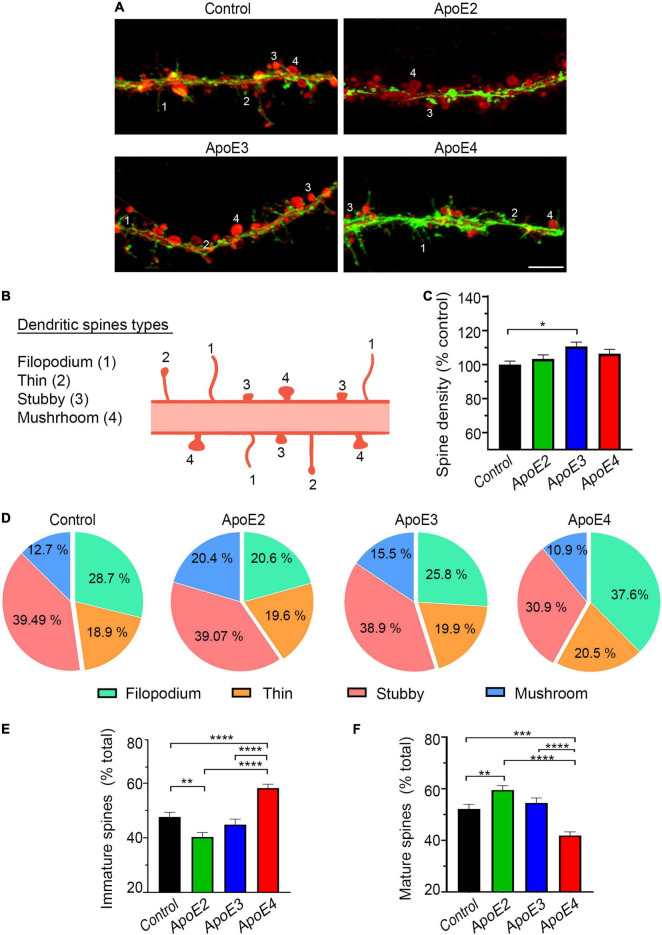
ApoE4 increases fraction of immature dendritic spines. **(A)** Representative microphotographs of dendritic spines in 17 DIV primary hippocampal neurons treated with the control conditioned media from *Apoe^–/–^* astrocytes or indicated apoE lipoproteins, which were stained with Phalloidin 488 (green) and DiI (red). Numerical labels denote types of dendritic spines 1 – filipodia, 2 – thin, 3 – stubby, and 4 – mushroom, which are schematically represented in **(B)**. Filipodia and thin spine represent immature spine forms and stain stronger with Phalloidin, while stubby and mushroom spines exemplify more mature spine forms and label more intensely with Dil. **(C)** Shown is analysis of dendritic spine density in 17 DIV hippocampal neurons treated with the control media or indicated apoE lipoproteins. Values are expressed relative to those in the control neurons and represent mean + SEM from 40 neurons per each treatment condition (10 neurons from each of four independent cultures). **(D)** Frequency analysis of spine types per treatment condition and the representation of immature (filipodia + thin) **(E)** and mature spines (stubby + mushroom) **(F)** as the fraction of the total spine number. Values represent mean + SEM (in **E,F**) from 40 neurons per treatment condition. Dendritic spine density remains relatively unchanged across the treatment conditions apart from a modest increase in apoE3 treated neurons. Analysis of spine type distribution shows that apoE2 significantly increases the fraction of mature spines at the expense of immature spines compared to the control neurons. ApoE4 significantly increases the fraction of immature spines and significantly decreases that of mature spines compared to all other treatment conditions. **(C)** *p* < 0.01, **(E)** *p* < 0.0001, and **(F)** *p* < 0.001 one-way ANOVA; *p < 0.05, **p < 0.01, ***p < 0.001, and ****p < 0.0001 (Holm’s–Sidak’s *post hoc* test). Scale bar 5 μm in **(A)**.

Dendritic spines were labeled with a combination of 1, 1′-Dioctadecyl-3, 3, 3′, 3′-tetramethylindocarbocyanine perchlorate (DiI) and Phalloiding-iFluor 488 (Abcam Inc., Cambridge, MA, United States), which have complementary labeling affinity toward mature and immature spine forms, respectively. Dil solution was prepared by dissolving Dil crystals in dimethyl sulfoxide (DMSO) (2 μg/μl), and then diluting the DMSO based solution with Dulbecco’s phosphate-buffered saline (DPBS) 1:500. Neurons were first fixed with 2% PFA and then stained with Dil overnight at room temperature. On the following day, after copious washing with DPBS the neurons were stained again with 1:1,000 PBS solution of Phalloidin-iFluor 488 for 90 min at room temperature (Cheng et al., 2014). Dil/Phalloidin-iFluor 488 double labeled neurons were imaged using Zeiss 880 LSCM with ZEN Black 2.3 SP1 acquisition software. Z-stack of images were acquired through the entire thickness of a neuron with a capture speed of 5, 54% overlap between slices, and a pinhole diameter of 1 Airy unit. The Z-stacks were collapsed into two-dimensional images using the maximum intensity projection function. The background was subtracted with a rolling ball radius of 35 pixels, gamma was adjusted to 1.1, and the resolution of the pictures was proportionally increased by a factor of 5 both in *X*- and *Y*-axis using the Transform J plugin in FIJI software. The total number and subtype frequency of dendritic spines were analyzed along four 20-μm long segments randomly selected along secondary independent dendrites. Four spine subtypes were recognized based on their distinct morphology: filopodium, thin, stubby, and mushroom-shaped (Cheng et al., 2014) (Figures 4A,B). Ten randomly selected neurons per culture from four independent cultures per treatment condition were analyzed.

### Synaptic Protein Expression

Primary hippocampal neurons were fixed 4% PFA and immunostained against postsynaptic density protein 95 (PSD95) with a mouse mAb (1:100; MilliporeSigma), NR1 subunit of the *N*-methyl-D-aspartate receptor (NMDAR) with a rabbit mAb (1:100; MilliporeSigma), or against synaptophysin with a mouse mAb (1:250; Santa Cruz Biotechnology, Dallas, TX, United States). Biotinylated anti-mouse or anti-rabbit secondary antibodies (both 1:500; Vector Laboratories) followed by Cy3 conjugated streptavidin (1:1,000) were used to visualize immunolabeled synaptic proteins. Neurons were imaged using Zeiss 880 laser scanning confocal microscope (LSCM) and ZEN Black 2.3 SP1 acquisition software (v. 14.0.18.201) (Carl Zeiss AG, Oberkochen, Germany) under the 63 × 1.4 N.A. objective. Z-stack of images were acquired through the entire thickness of the neuron with a capture speed of 7, 67% overlap between slices, and a pinhole diameter of 1 Airy unit. To quantify expression of synaptic proteins Z-stacks were collapsed into a two-dimensional image, which was converted into 8-bit format. Expression of synaptic proteins was quantified along secondary independent dendrites following our previously published protocols (Kuszczyk et al., 2013, 2014). Ten to 15 neurons per culture from four independent cultures per treatment condition were randomly chosen for analysis. Five rectangular test areas (20 μm × 10 μm) covering secondary independent dendrites were selected per neuron. Integrated density of the synaptic protein immunostaining within each test area was then measured using the FIJI software. The integrated density is defined as a product of area and mean gray value for a positive immunostaining within the test area.

### RNA Isolation and Deep Sequencing

Neuronal RNA was isolated using RNeasy Mini Kit, following manufacturer provided protocol, which included in column DNA digestion with 2 U of DNAse I. Processing of isolated RNA and its sequencing was carried out by the Genome Technology Center of the NYU Grossman School of Medicine. Purity and integrity of isolated RNA was first determined using 2100 Bioanalyzer (Agilent Technologies Inc., Santa Clara, CA, United States). RNA concentration was measured on NanoDrop 2000 spectrophotometer (Thermo Fisher Scientific). Ribosomal RNA was depleted using the Ribo-Zero Gold kit (Illumina, Inc., San Diego, CA, United States). cDNA libraries were generated with Illumina Truseq kit with an input of 200 ng of RNA and 10 cycles. Sequencing was performed using the NovaSeq 6000 Sequencing System on an SP100 flowcell (Illumina, Inc.).

### Bioinformatic Analysis of the Transcript

Analysis of RNA sequencing readouts was performed with the help of the Applied Bioinformatics Laboratory of the NYU Grossman School of Medicine. Sequencing reads were mapped to the reference genome (mm10) using the STAR aligner software (v2.5.0c) (Dobin et al., 2013). Alignments were guided by a Gene Transfer Format (GTF) file. The mean read insert sizes and their standard deviations were calculated using Picard tools software (v.1.126)^1^. The read count tables were generated using HTSeq software (v0.6.0) (Love et al., 2014) and normalized based on their library size factors using the DEseq2 package (Quinlan and Hall, 2010). The gene expression analysis aimed to identify genes enriched by each apoE isoform compared to the control where apoE was absent. The Read Per Million normalized BigWig files were generated using BEDTools software (v2.17.0) 33 and bedGraphToBigWig tool (v4). Sets of genes enriched by each apoE isoform were analyzed using Kyoto Encyclopedia of Genes and Genomes (KEGG) with the aid of the DAVID Functional Classification Tool^2^ (Huang da et al., 2009). This allows for identification of pathways enriched by each apoE isoforms. Significantly up and down regulated genes, which are not included in any recognized KEGG pathway, were ontogenetically classified (gene ontology [GO] analysis) based on the function of its coded protein included in the Uniport database (UniProt, 2019) and/or by literature review for their specific roles in neuronal biology. Genes were considered differentially expressed if their RNA level changed at least two fold with *p* < 0.05 (Wang et al., 2021; Yamaguchi et al., 2021) between any of the apoE isoform treatment and the control neurons grown in the absence of added human apoE lipoproteins. Four independent neuronal cultures per each apoE isoform and four control cultures grown in the apoE absence were subjected to RNA sequencing and subsequent bioinformatic analysis.

### Data Presentation and Statistical Analysis

All data sets characterizing primary hippocampal neurons cultured in the presence of apoE lipoproteins are expressed relative to the control neurons grown in the presence of conditioned media from *Apoe^–/–^* astrocyte. All data sets were first assessed for normality using Shapiro–Wilk test. Differences across various treatment conditions were analyzed using one-way analysis of variance (ANOVA) except for comparison of dendritic arborization (Sholl’s analysis) where two-way ANOVA was used. Holm–Sidak test was chosen for *post hoc* analysis for one- and two-way ANOVA. All ANOVA analyses were performed using GraphPad Prism v8.4.3 (GraphPad Software Inc., San Diego, CA, United States). Differences in the individual gene expression between neurons treated with each of apoE lipoproteins and the control neurons were analyzed using Student’s *t-test*, which was performed in the R environment (v3.1.1)^3^. To determine the significance of KEGG pathway enrichment Fisher’s exact test was used and the analysis was performed using DAVID Functional Classification Tool. All data in the manuscript are presented as the mean and the standard error of the mean (SEM).

## Results

### Characterization of ApoE Lipoprotein Particles and Their Uptake by Hippocampal Neurons

Western blotting analysis of gels run under native conditions confirmed production of lipidated apoE particles by immortalized astrocytes and their secretion to the conditioned media (Figures 1A, 2A,B). Most of the apoE2 and apoE3 lipoproteins are large particles, which under native conditions resolve above the 242 kDa marker: 83.3% ± 4.7 and 91% ± 2.7%, respectively. Small particles, which resolve below the 242 kDa marker constitute only 18.6% ± 3.3 and 10.7% ± 4.5% of the total apoE2 and apoE3 particle pool, respectively. The relationship between large and small particles for apoE4 is inversed. The former constitutes only 35.9% ± 6.5% of the total apoE4 particle pool, while the later 59.7% ± 8.7%. These noticeable disparities in lipidated apoE particle size previously described in the literature underscore differences in the lipid binding characteristics across various apoE isoforms, indicating relatively poorer lipidation of the apoE4 (Morikawa et al., 2005; Kim et al., 2015; Fu et al., 2016; Chen Y. et al., 2021).

We confirmed that at the commencement of the experiment, the level of apoE protein in the neuronal media is similar for all apoE isoforms (Figures 2C,E). It averages 41.6 ng/mL ± 2.6 ng/mL as measured by the apoE ELISA (Figure 2E). This apoE concentration is comparable to the apoE concentration in the brain interstitial fluid of *APOE* ε3/ε3 targeted replacement mice, which is estimated at 38.1 ng/mL ± 4.0 ng/mL using *in vivo* microdialysis zero-flow method (Ulrich et al., 2013). We also directly confirmed internalization of apoE by neurons. At the conclusion of the experiment presence of the apoE protein in the neuronal lysate is both detectable by Western blotting of gels resolved under reducing conditions and ELISA (Figures 2D,F). Neurons grown in the presence of apoE4 contain 30.68% more apoE protein than neurons grown in the presence of apoE2 (*p* < 0.05) and 20.84% more than those grown in the presence of apoE3 (*p* = 0.067) (Figure 2F). The difference between apoE2 and apoE3 cultured neurons is negligible.

We also confirmed *Apoe^–/–^* astrocytes secrete no apoE to the conditioned media. The control neurons treated with the conditioned media from *Apoe^–/–^* astrocytes show no detectable apoE protein both in the neuronal media and in the neuronal lysate (Figure 2).

### ApoE Isoforms Differentially Regulate Complexity and Extent of the Dendritic Arbor

Our experimental setup shows that apoE isoforms differentially regulate complexity and extent of the dendritic arbor in primary hippocampal neurons cultured *in vitro*. Neurons, which between 10 and 17 DIV were cultured in the presence of apoE2 or apoE3 lipoproteins show a significant increase in the number of dendritic branches compared to the control neurons grown in the absence of added human apoE lipoproteins (Figure 3A). Sholl’s analysis reveals the greatest difference in the number of dendritic branches in the range of 30–110 μm from the soma, thus concerning primary and secondary dendrites (*p* < 0.0001 apoE2 vs. control and *p* < 0.0001 apoE3 vs. control) (Figure 3C). ApoE2 and apoE3 lipoproteins also increase the combined dendrite length by 10.8% ± 6% (*p* = 0.14) and 17.2% ± 6% (*p* < 0.05) compared to the control, respectively; and the total arbor surface by 26% ± 8.3% (*p* < 0.01) and 24.7% ± 8.3% (*p* < 0.01), compared to the control, respectively (Figures 3D,E). There are no significant differences concerning the number of dendritic branches, the combined dendrite length, and the total arbor surface between neurons cultured in the presence of apoE2 or apoE3 lipoproteins.

Neurons grown in the presence of apoE4 lipoproteins show significantly lower number of dendritic branches than those cultured in the presence of apoE2 (*p* < 0.0001) or apoE3 (*p* < 0.0001) lipoproteins. The difference in the number of branches between apoE4 treated neurons and the control neurons also reach statistical significance (*p* < 0.05) but this is more likely a result of the sum of minor variances in the number of cross points at various concentric circles, rather than represents any systematic effect attributable to apoE4 (Figures 3A,C). ApoE4 treated neurons demonstrate significantly lower values of the combined dendrite length and the total arbor surface metrics compared to those treated with apoE2 and apoE3 (Figures 3D,E). In comparison to the control neurons apoE4 reduces the combined dendritic length by 16% ± 6% (*p* < 0.05) but does not change the total arbor surface.

### ApoE Isoforms Show Opposing Effects on Dendritic Spine Subtype Frequency

Formation and maturation of the dendritic spines are the mechanisms underlying neuronal network plasticity. ApoE lipoproteins show no systematic effect on the dendritic spine density compared to the control neurons. Only apoE3 modestly increases the density of spines by 11.1% (*p* < 0.05) (Figures 4A,C). There is, however, apoE isoform specific effect on the subtype frequency of dendritic spines. We distinguished four forms of the dendritic spines listed in the order of advanced maturation and readiness to form the synaptic connections: filopodium, thin, stubby, and mushrooms (Figures 4A,B). In the control neurons grown in the absence of added human apoE lipoproteins the percentages of immature spines (filopodium + thin) and mature spines (stubby and mushroom) constitute 47.62% ± 1.7% and 52.1% ± 1.8%, respectively (Figures 4D–F). ApoE2 significantly increases the fraction of mature spines to 59.47% ± 1.74% (*p* < 0.01) at the expense of immature spine, which fraction is reduced to 40.3% ± 1.63% (*p* < 0.01) compared to the control. Similar trend, yet statistically insignificant vs. the control, is seen for apoE3, which increases the fraction of mature spines to 54.4% ± 1.95% (*p* = 0.34), while reducing the fraction of immature spines down to 44.8%, ± 2.0% (*p* = 0.24). In comparison, apoE4 lipoproteins produce an opposite effect increasing the fraction of immature spines to 58.2% ± 1.4% (*p* < 0.0001) and reducing the fraction of mature spines to 41.9% ± 1.4% (*p* < 0.001) compared to the control. The differences in the spine fractions between neurons treated with apoE4 lipoproteins and those treated with apoE2 or apoE3 lipoproteins also are significant (Figures 4D–F).

### ApoE Isoforms Show Opposing Effects on the Dendritic Expression of PSD95 and NR1 Synaptic Proteins

**FIGURE 5:**
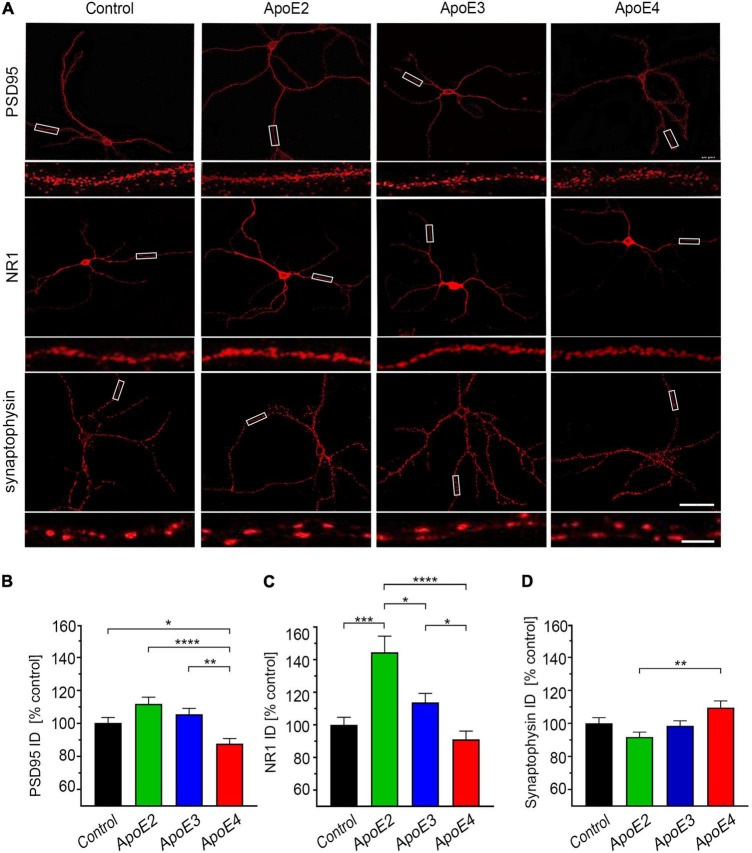
ApoE isoforms differentially regulate expression of PSD95 and NR1. **(A)** Representative microphotographs of 17 DIV hippocampal neurons grown in the presence of the control media from *Apoe^–/–^* astrocytes or indicated apoE particles, which were immunostained against post-synaptic density protein 95 (PSD95), NR1 subunit of the *N*-methyl-D-aspartate receptor or synaptophysin. Rectangular areas covering section of dendrites are enlarged directly underneath each image and exemplify test areas used to analyze integrated density of synaptic proteins. Shown is analysis of the integrated density for PSD95 **(B)**, NR1 **(C)**, and synaptophysin **(D)** in the independent secondary dendrites. Values are expressed relative to those in the control neurons and represent mean + SEM from 40 to 60 neurons per each treatment condition (10–15 neurons from each of four independent cultures). ApoE4 treated neurons show significantly reduced expression of PSD95 compared to all other treatment conditions and the NR1 expression compared to apoE2 and apoE3 treated neurons but not to the control neurons. ApoE2 significantly increases NR1 expression of compared all other treatment conditions. **(B,C)** *p* < 0.0001 (ANOVA); *p < 0.05, **p < 0.01, ***p < 0.001, and ****p < 0.0001 (Holm’s–Sidak’s *post hoc* test). Scale bar 40 and 5 μm (inset) in **(A)**.

ApoE2 and apoE3 lipoproteins increase expression of PSD95 and NMDAR NR1 subunit in the dendrites. The integrated density of PSD95 is higher by 11.8% ± 4.9% (*p* = 0.064) and 5.6% ± 4.9% (*p* = 0.38) in apoE2 and apoE3 treated neurons, respectively; while that of NR1 by 33.2% ± 8.0% (*p* < 0.0001) and 11.7% ± 8.1% (*p* = 0.27), respectively (Figures 5A–C). In contrast, apoE4 lipoproteins reduce the integrated density of PSD95 by 12.5% ± 4.9% (*p* < 0.05) and show a parallel trend to reduce that of NR1 by 9.9% ± 8.2% (*p* = 0.27) compared to the control neurons. Due to opposing direction of changes *post hoc* differences in PSD-95 and NR1 integrated density values between neurons treated with apoE4 and those treated with apoE2 or apoE3 are significant (Figures 5A–C). No apoE isoform significantly alters expression of synaptophysin compared to the control neurons (Figure 5D). A statistically significant *post hoc* effect is observed between apoE2 and apoE4 treated neurons (*p* < 0.01) due to opposing direction of change in the synaptophysin integrated density values with the former showing 8.3% ± 4.7% (*p* = 0.20) reduction and the later 9.5% ± 4.8% (*p* = 0.18) increase compared to the control neurons.

### ApoE Isoforms Differentially Enrich Neuronal Transcriptome

**FIGURE 6:**
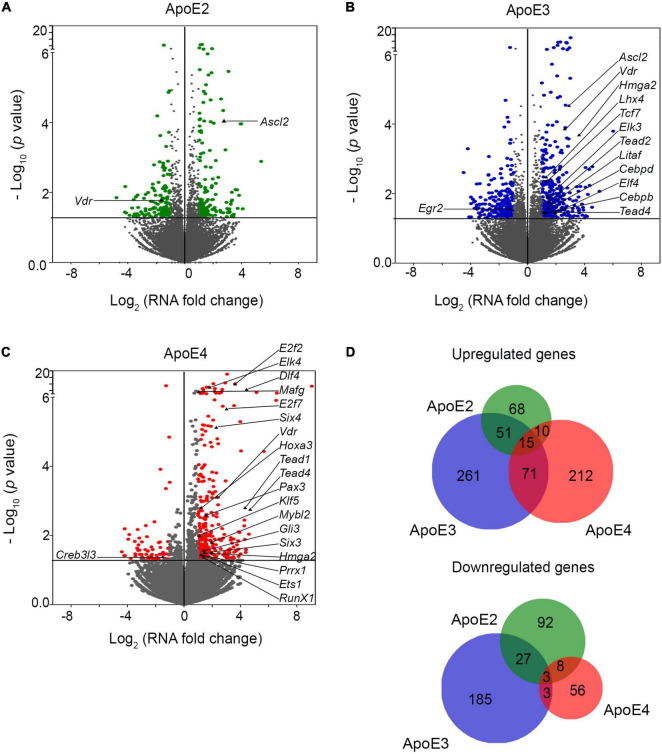
ApoE lipoproteins differentially modulate transcriptomic profile of maturating hippocampal neurons. Panels **(A–C)** show volcano plots of genes, which are enriched in primary hippocampal neurons maturated in the presence of indicated apoE lipoproteins compared to the control neurons grown in the presence of conditioned media from *Apoe^–/–^* astrocytes. Abscissas represent Log_2_ of the mRNA level fold change vs. the control neurons and ordinates represent negative Log_10_ of *p*-values for the genes variably expressed under a given apoE lipoprotein treatment condition. The horizontal lines indicate the *p*-value of 0.05. Genes, which *p*-value is less than 0.05 and mRNA fold change is greater than two are considered significantly enriched and are highlighted in color. Significantly enriched transcriptomic factors are indicated by their names. ApoE3 effects the greatest number of enriched genes while the apoE2 the smallest. **(D)** Shown are the Venn diagrams depicting the overlap of genes, which are enriched by the treatment with various apoE lipoproteins. Gene enrichment effect is relatively apoE isoform specific. There is a moderate overlap between gene sets enriched by apoE2 and apoE3, and those enriched by apoE3 and apoE4, while the overlap between apoE2 and apoE4 and across all three apoE isoforms is negligible.

Analysis of neurons grown in the presence of lipoproteins comprised of various apoE isoforms shows a substantial transcriptomic enrichment compared to the control neurons grown in the absence of added human apoE lipoproteins (Figures 6A–C). The observed effect is largely apoE isoform specific as the overlap between gene sets enriched by two or more of apoE lipoproteins is moderate (Figure 6D). The most robust effect is caused by apoE3 which upregulates expression of 398 genes and downregulate expression of 218. ApoE4 upregulates expression of 308 genes and down regulates expression of 70 genes. ApoE2 produces the least robust effect upregulating 144 genes and down regulating 130 genes. There is a moderate overlap between genes differentially enriched by apoE3 and apoE2 with 51 and 27 commonly up and down regulated, respectively; and between apoE3 and apoE4 with 71 and 3 commonly up and down regulated, respectively. In contrast, the number of genes commonly up and down regulated by apoE2 and apoE4 are only 10 and 8, respectively. Out of the pool of 1,062 genes differentially enriched by any of apoE isoforms only 15 are upregulated and 3 are downregulated by all three isoforms.

There are numerous transcription factors among genes, which are differentially enriched by apoE lipoproteins (Figures 6A–C and Supplementary Table 1). Interestingly, the most robust effect is caused by apoE4, which affects expression of 20 transcription factors. ApoE3 alters expression of 12 while apoE2 only two. Most of these differentially expressed transcription factors are upregulated. Only *Egr2* is downregulated by apoE3, while apoE4 downregulates *Creb3l3*. There is a minimal overlap across transcription factors enriched by various apoE isoforms. Only *Vdr* encoding vitamin D3 receptor is upregulated by all three of them. About 40% of transcription factors, which expression is affected by various apoE lipoproteins also belong to KEGG pathways, significantly enriched by the apoE lipoproteins.

### Signaling Pathways and Gene Sets Enriched by ApoE4 Lipoproteins Are Functionally Distinct From Those Enriched by ApoE2 and ApoE3 Lipoproteins

**FIGURE 7:**
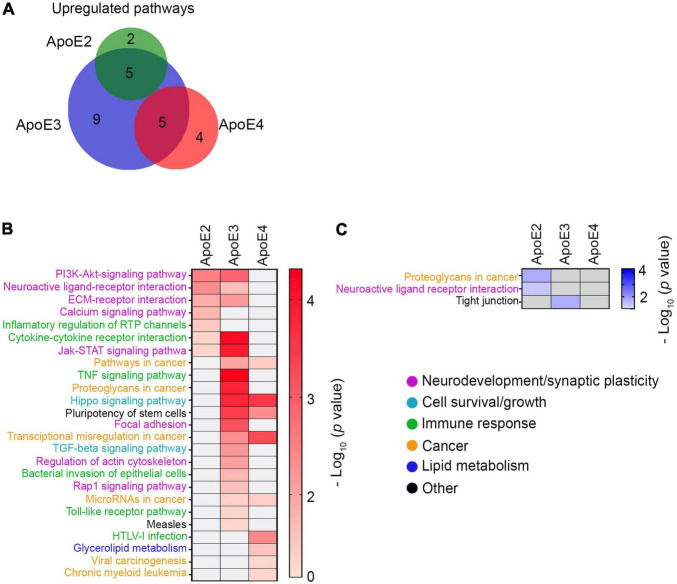
KEGG pathway analysis. **(A)** Shown is a Venn diagram depicting the overlap of KEGG pathways differentially upregulated by various apoE lipoproteins. While there is a considerable overlap between the number of KEGG pathways upregulated by apoE2 or apoE3 and those upregulated by apoE3 or apoE4, there is no overlap between apoE2 and apoE4 and across all three apoE isoforms. Shown are heat maps of KEGG pathways, which are significantly upregulated **(B)** or downregulated **(C)** in neurons maturated in the presence of indicated apoE lipoproteins vs. the control neurons. Color intensity gradients included alongside each heatmap graphically represent negative Log_10_ of *p*-values for enriched KEGG pathways.

KEGG pathway analysis shows apoE3 lipoproteins upregulate the greatest number of pathways 19, while apoE4 and apoE2 lipoproteins upregulate 10 and 7 pathways, respectively (Figures 7A,B and Supplementary Table 2). There are five pathways commonly upregulated by apoE3 and apoE2 and another 5 by apoE3 and apoE4, but there is no single pathway, which is commonly upregulated by apoE2 and apoE4 or by all three apoE isoforms. Relatively few pathways are down regulated. ApoE2 down regulates two pathways, while apoE3 down regulates one (Figure 7C and Supplementary Table 2).

KEGG pathways enriched by various apoE isoforms are functionally categorized as related to (1) neurodevelopment and synaptic plasticity, (2) cell survival and growth, (3) immune response, (4) cancer, (5) lipid metabolism, and (6) others. Pathways upregulated by apoE2 and apoE3 are functionally distinct from those primarily upregulated by apoE4. Both apoE2 and apoE3 but not apoE4 significantly upregulate four signaling pathways importantly involved in neurodevelopment and synaptic plasticity: PI3-AKT pathway (*p* < 0.01 apoE2; *p* < 0.01 apoE3), neuroactive ligand-receptor pathway (*p* < 0.01 apoE2; *p* < 0.05 apoE3), ECM receptor interaction pathway (*p* < 0.05 apoE2; *p* < 0.01 apoE3) and JAK-STAT pathway (*p* < 0.05 apoE2; *p* < 0.001 apoE3). ApoE2 also exclusively upregulates the calcium signaling pathway (*p* < 0.05), while apoE3 the focal adhesion pathway (*p* < 0.001) and the actin cytoskeleton regulatory pathway (*p* < 0.01), and in the category of pathways involved in cell survival and growth the TGFβ signaling pathway (*p* < 0.01). ApoE2 has down regulating effect on a part of the neuroactive ligand-receptor pathway (*p* < 0.05) (Figure 7C). ApoE4 does not upregulate any pathways classified as related to neurodevelopment and synaptic plasticity either exclusively or in common with other apoE isoforms. It does upregulate commonly with apoE3 the Hippo signaling pathway, which is related to cell survival and growth (*p* < 0.001 apoE3; *p* < 0.001 apoE4). ApoE4 also upregulates pathways involved in glycerophospholipid metabolism (*p* < 0.05). All types of apoE isoforms upregulate, either commonly or individually, several pathways related to cancer and immune response, which implication in the biology of developing neurons is unclear (Figure 7B).

**FIGURE 8:**
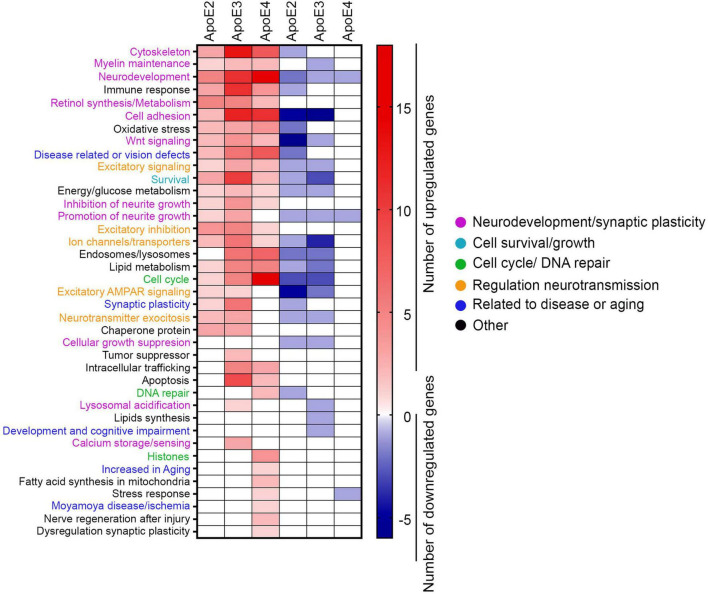
GO analysis. The heat map depicts the number of genes for each of the ontological category, which are differentially enriched by indicated apoE lipoprotein treatment compared to the control neurons. Only genes showing twofold change and *p*-value < 0.05 are represented.

There are numerous genes differentially enriched by treatment with various apoE lipoproteins, which do not belong to any established KEGG pathway. We subjected these genes to the GO analysis according to the function their encoded proteins play in neurons. Protein function was cross-referenced against the Uniprot database and the literature search (Figure 8 and Supplementary Table 3). All apoE isoforms commonly enrich a category of genes pertinent to cytoskeleton structure and maintenance, myelin maintenance, neurodevelopment, immune response, synthesis and metabolism of retinol, cell adhesion, oxidative stress, Wnt signaling, excitatory signaling, neurological and sensory disorders, excitatory signaling, survival, and energy and glucose metabolism. In categories including neurite growth control, excitatory inhibition, and ion channels, transporters genes are predominantly enriched by apoE2 and apoE3 with few genes affected by apoE4, while genes involved in exosomal and lysosomal biology, lipid metabolism and cell cycle are largely enriched both by apoE3 and apoE4 and to lesser extent by apoE2. Genes involved in the excitatory α-amino-3-hydroxy-5-methyl-4-isoxazolepropionic acid receptor (AMPAR) signaling, synaptic plasticity, neurotransmitter exocytosis, chaperone proteins, and suppression of cellular and tumor growth are enriched by apoE2 and apoE3 but not by apoE4. ApoE3 and apoE4 but not apoE2 affect expression of genes involved in intracellular trafficking, apoptosis, DNA repair. There is a prominent group of genes exclusively enriched by apoE4, which includes histone structural proteins.

## Discussion

Previously several groups including ours have shown that apoE promotes dendritic arborization, dendritic spine sprouting, synapse formation, and various synaptic protein expression (Gordon et al., 1995; Raber et al., 1998, 2000; Qiu et al., 2004; Kuszczyk et al., 2013; Kim et al., 2014). Each of these biological functions play an important role in the development, long-term maintenance, and adaptive plasticity of neuronal networks. In this paper we have shown native apoE lipoproteins differentially influence neuroplasticity mechanisms through epigenetic regulation of neuronal transcriptome. In primary hippocampal neurons treated with human apolipoproteins, which concentrations mimic that established in the brain interstitial fluid, apoE2 and apoE3 increase extent and complexity of the dendritic arbor while apoE4 show no effect on the number of dendritic branches and the total arbor surface compared to the control neurons, while it reduces the combined dendrite length. ApoE lipoproteins also regulate the frequency of various dendritic spine types in seemingly differing ways. ApoE2 and apoE3 increase the frequency of mature spines (i.e., stubby and mushrooms) at the expense of immature spines (i.e., filipodia and thin), while apoE4 produces an opposite effect by reducing the frequency of mature spines and increasing the frequency of immature spines. However, apoE lipoproteins show no systematic effect on dendritic spine density. Mature spines can readily form synaptic contacts and feature greater expression of PSD95 and NMDAR NR1 subunit (Hering and Sheng, 2001; Yoshihara et al., 2009), which we also noticed in apoE2 and apoE3 treated neurons. In contrast, immature spine types like filopodia, which are upregulated by apoE4, feature enhanced plasticity potential and can be rapidly formed or eliminated. They play an important role in fast one-trial learning (Ozcan, 2017). Interestingly, the work by Nwabuisi-Heath et al. (2014) also suggested that apoE4 may not only prevent dendritic spine maturation, but it also may precipitate loss of mature spines promoting dendritic spine turnover. In accordance with these observations, analysis of neuronal structure in young *APOE*-ε*4* targeted replacement mice revealed simpler neuronal network characteristics compared to *APOE*-ε*3* mice with key differences concerning reduced dendritic branching, density and length of dendritic spines (Dumanis et al., 2009; Rodriguez et al., 2013). These discrete effects caused by apoE4 appear to leave the neurons in a less mature state but endows them with a greater plasticity potential, should they engage in the formation of neuronal networks. In fact, several groups have independently noted the superior level of cognitive performance and in particular the enhancement of episodic memory in young *APOE*ε*4* carriers compared to non-carriers (Yu et al., 2000; Mondadori et al., 2007; Jochemsen et al., 2012; Rusted et al., 2013). As cognitive task performance relies on the adaptive plasticity of neuronal networks, reduced degree of dendritic spine maturation and its increased turnover featured by *APOE* ε*4* carriers render them more capable of adaptive changes during the cognitive tasks. Consistent with this notion, the enhancement of hippocampal LTP at younger age in *APOE*-ε*4* mice also was shown (Kitamura et al., 2004). The outstanding performance in *APOE ε4* carriers at young age remains in stark contrast to the effect of *ε4* allele during aging when it becomes a dominant risk factor for age related cognitive decline (Greenwood et al., 2000; Caselli et al., 2007, 2009, 2011; Luck et al., 2015). Opposing effects the ε*4* allele on cognition in young vs. aging carriers is known as the antagonistic pleiotropy effect (Tuminello and Han, 2011; Rusted and Carare, 2015; Di Battista et al., 2016). Mechanisms underlying worse cognitive outcomes in elderly carriers of the ε*4* allele remain obscure, but one can suggest detrimental effect of enhanced synaptic plasticity throughout the lifespan leading to accelerated senility (Nuriel et al., 2017a). Detrimental effect of the ε*4* allele on the aging of neuronal networks has been consistently reproduced in multiple transgenic mouse lines expressing human apoE4 under the control of various promoters, which showed both behavioral and LTP deficiencies with advanced age [reviewed in Kim et al. (2014)]. Though *APOE* ε*4* carriers remain at significantly greater risk for AD (Corder et al., 1993) due to increased propensity to develop β-amyloid (Castellano et al., 2011; Holtzman et al., 2012) and tau pathologies (Shi et al., 2017), detrimental, age-related effect of the *APOE* ε*4* allele on neuronal network plasticity potential constitutes an independent risk factor promoting earlier age of onset and more rapidly progressing AD symptoms due to limiting the cognitive reserve [reviewed in Yamazaki et al. (2019)].

The main conclusion drawn from the transcriptomic analysis of primary hippocampal neurons treated with human apoE lipoproteins, is that the effect of the apoE lipoproteins is not systemic but rather isoform specific with relatively moderate overlap between gene sets commonly enriched by two or more isoforms. Importantly this moderate overlap between KEGG pathways and ontological gene categories exists between apoE2 and apoE3 and between apoE3 and apoE4. In contrast, the overlap between apoE2 and apoE4 and likewise between all three apoE isoforms is minimal. From the functional perspective effects of transcriptomic enrichment caused by apoE2 and apoE3 are often similar, while those caused by apoE3 and apoE4 are at times oppositional. Both apoE2 and apoE3 commonly upregulate PI3K-AKT, neuroactive ligand receptor pathway, ECM-receptor interaction, and Jak-STAT signaling pathways where numerous genes are involved in promoting neurite branching (Peterson et al., 2010; Kerrisk et al., 2014; Skupien et al., 2014; Woodbury and Ikezu, 2014; Roszkowska et al., 2016; Kurshan and Shen, 2019), long term potentiation (McLean et al., 1993; Evers et al., 2002; Rosch et al., 2005; Gratacos et al., 2009; Guimond and Gallo-Payet, 2012; Daimon et al., 2013; Gross and Bassell, 2014; Garcia-Recio and Gascon, 2015; Linden et al., 2018; Sherman and Bang, 2018), or inhibitory signaling (Becker et al., 2006; Horvath et al., 2014; McCracken et al., 2017). In addition apoE2 and apoE3 also enrich multiple genes involved in retinoic acid metabolism and calcium signaling, which also are implicated in cellular maturation and neuronal signaling (Chen W. et al., 2010; Iwasawa et al., 2012; Sheng et al., 2013; Chou and Wang, 2016; Stoney et al., 2016). In contrast, apoE4, which has opposite effects on dendritic arbor and spine subtype characteristics shows less conspicuous effect on the expression of genes involved in retinoic acid and calcium metabolism. Instead apoE4 strongly upregulates genes involved in glycolipid turnover including *Prkd3*, which increases diacylglycerol (DAG) signaling, involved in dendritic spines destabilization (Kim et al., 2010; Buchser et al., 2012). Thus, activation of DAG signaling could be contributory to the higher number of immature dendritic spines observed in neurons treated with apoE4 lipoproteins. ApoE4 also upregulates *Hipk2*, which suppresses the expression of NMDAR subunits and this effect could be directly related to reduced expression of NR1 and PSD95 which is functionally and anatomically associated with the NMDAR (Shang et al., 2018).

Both apoE2 and apoE3 also enrich genes involved in eliminating extra synapses, such as *C1ql1* and genes that suppress cell growth, which suggests other ways these apoE isoforms regulate synaptic plasticity and control excessive synaptogenesis to maintain only essential, or mature, connections between neurons. ApoE2 and apoE3 commonly affect excitatory signaling mediated by AMPARs receptors by increasing expression of *Syndig1l* and downregulating *Gsg1l* which underlies increased AMPAR expression (Kalashnikova et al., 2010; Gu et al., 2016). In addition, their downregulation of *Nptx1*, *Wnt7a* and *Gpc3* suggests increased control of excessive excitatory signaling by AMPAR (DeGregorio-Rocasolano et al., 2001; Figueiro-Silva et al., 2015; Schaukowitch et al., 2017). ApoE2 and apoE3 also commonly upregulate genes encoding ion channel proteins, which are involved in hyperpolarization and depolarization of neurons (Sugita et al., 2002; Lewerenz et al., 2013; Andero et al., 2014; Zhang et al., 2015; Kheradpezhouh et al., 2017; Polster et al., 2018; Rahmati et al., 2018) and enhancement of excitatory activity. Examples of these genes include *Otof* and *Stx11* or *Slc30a3* (Qian and Noebels, 2005; Johnson and Chapman, 2010; Martel et al., 2010; Zhou et al., 2013). On the other hand, regulatory effects of apoE2 and apoE3 seem to prevent hyperexcitability by downregulation of genes involved in neurotransmitter release and upregulation of genes related to enhancement of inhibitory signaling by GABA, galanin and glycine (Levanon et al., 2002; Becker et al., 2006; Anselmi et al., 2009; Horvath et al., 2014; McCracken et al., 2017; Tao et al., 2018). These observations suggest apoE2 and apoE3 regulate both excitatory and inhibitory hippocampal inputs maintaining compensatory balance between them. In contrast, apoE4 affects expression of several genes related to excitotoxicity including *Slc7a11* (Bridges et al., 2012) (also upregulated by apoE3) and *Gpr39* (Gilad et al., 2015) but does not enrich genes involved in the inhibitory input. Hyperexcitability and swift synaptic plasticity along with decreased background synaptic inhibition have been described in *APOE ε4* targeted replacement mice lacking AD pathology and have been suggested as causative for memory and learning impairment exhibited by these mice (Persson et al., 2008; Andrews-Zwilling et al., 2010, 2012; Tong et al., 2016; Nuriel et al., 2017a). Mechanism underlying this phenomenon, which has potential implication in accelerating cognitive aging and increased AD susceptibility among *APOE ε4* carriers is unclear. It has been explained by the loss of GABA-ergic neurons loss due to the presumed toxicity of apoE4 expressed by these neurons (Knoferle et al., 2014). However, our transcriptomic data establish new evidence to suggest apoE lipoproteins directly regulate neuronal excitatory potential, which is augmented in the presence of apoE4 and balanced out by the other isoforms. This more balanced regulatory effects of apoE2 and apoE3 on excitatory and inhibitory signaling, appears to be essential for neuroprotection and cognition maintenance (Persson et al., 2008; Andrews-Zwilling et al., 2010, 2012; Tong et al., 2016; Nuriel et al., 2017a).

Though apoE3 and apoE4 lipoproteins enrich a common set of KEGG pathways and ontological gene categories the functional effect of this enrichment in many cases may be different or even opposing. Both apoE3 and apoE4 enrich genes related to lipid metabolism in neurons. ApoE3 downregulates *Lipg* implicated in glutamate signaling and upregulates *Liph*, which is involved in neurite branching, what is consistent with the observed effect of apoE3 on enhancing dendritic tree complexity (Yung et al., 2015; Roza et al., 2019; Yun et al., 2019). ApoE4 upregulates *AloX8*, which is involved in lipid signaling and *Apobr*, which encodes the apolipoprotein B receptor. Previous reports showed that interaction of apoE4 with neuron expressed apoE receptor 2 decreases the availability of this receptor at the neuronal membrane (Chen Y. et al., 2010; Xian et al., 2018), hence upregulation of *Apobr* could constitute a mechanism to increase the availability of apoE receptor 2 and also to help lipid internalization especially that apoE4 lipoproteins contain significantly less lipids compared to lipoproteins comprised of apoE2 and apoE3 (Barber and Raben, 2019).

The Hippo signaling pathway is an evolutionarily conserved pathway that controls cell proliferation, apoptosis, and stem cell self-renewal (Lavado et al., 2018). Our transcriptomic studies provide novel evidence that both apoE3 and apoE4 strongly enrich the Hippo pathway during neuronal maturation, however, effects of apoE3 and apoE4 appear to be opposing. Whilst apoE4 upregulates genes related to the Hippo pathway activation the apoE3 upregulates genes involved in its inhibition. Activation of the Hippo pathway has been associated with the transcription of genes involved in homeostasis and neuroprotection, however, its hyperactivation may be deleterious, triggering DNA damage and apoptosis. Hyperactivation of the Hippo pathway also is linked to higher susceptibility to cellular death and progression of neurodegeneration (Wang and Wang, 2016).

ApoE3 and apoE4 also commonly regulate genes related to endosomal and lysosomal pathway, suggesting higher endocytosis of receptors or increased neurotransmitter recycling (Gindhart and Weber, 2009; Lee et al., 2011, 2012; Kam et al., 2013; Breusegem and Seaman, 2014). Previous studies showed that apoE4 alters the endosomal-lysosomal pathway and produces an enlargement of endosomal-lysosomal compartments (Nuriel et al., 2017b). Consistently, our studies showed increased intraneuronal retention of apoE4 compared to apoE2 and apoE3 isoforms, which may lead to upregulation of transcript related to endosomal and lysosomal pathway.

Several ontological gene categories appeared to be selectively enriched in neurons maturated in the presence of apoE4 lipoproteins. These include genes involved in cell cycle, DNA repair and histones and their expression is usually associated with cellular stress (Fishel et al., 2007; Szpara et al., 2007; Greenberg, 2008; Wang et al., 2009; Arendt et al., 2010; Frade and Ovejero-Benito, 2015). Additionally, apoE4 induces expression of *Trib3* involved in neuronal apoptosis (Zhang et al., 2019). ApoE2 and apoE3 but not apoE4 upregulate genes expressing heat shock protein and other protein folding chaperones, which protect neurons from aggregation and accumulation of misfolded proteins as this is often a leading pathological event in several neurodegenerative diseases (Galvin et al., 2006; Srivastava et al., 2012; Crippa et al., 2016; Ousman et al., 2017). Heat shock proteins also help to stabilize cytoskeleton and prevent apoptosis (Leak, 2014; Treweek et al., 2015; San Gil et al., 2017). These observations imply that apoE isoforms differentially regulate neuronal transcriptomic profile already during maturation rendering it more or less predisposed to neurodegeneration.

There are several ways through which apoE lipoproteins may exert their regulatory effect on neuronal transcriptome. Most commonly accepted mechanisms include differential interaction with neuron-expressed apoE receptors and subsequent engagement of intraneuronal signaling pathways (Qiu et al., 2004; Chen W. et al., 2010; Holtzman et al., 2012; Zhao et al., 2018). It also has been suggested that apoE lipoproteins may contain RNA species of astrocytic origin and those upon the lipoprotein internalization may regulate neuronal lipid metabolism (Li et al., 2021). Our work provides evidence that apoE lipoproteins enrich multiple neuronal transcription factors and that this effect is the strongest for apoE4, which also shows the greatest intraneuronal accumulation. Thus, it is possible that upon internalization apoE directly engages regulatory mechanism of the neuronal transcriptome. Previous work has identified a specific apoE4 DNA binding sequence while subsequent genome-wide mapping indicated its presence across ∼1,700 gene promoter regions (Theendakara et al., 2016). The direct transcriptional effect of apoE4 has been postulated to occur during AD pathogenesis owing nuclear translocation of apoE4 liberated from ruptured endosomal vesicles (Theendakara et al., 2018). This work suggests the interaction between apoE and the neuronal DNA may play a role in physiological neuronal functions. At the moment it remains unclear to what extent epigenetic effects of apoE lipoproteins depend on differences in their lipid content (Chen Y. et al., 2021). Our experimental setup, which assumed neuronal treatment with equimolar concentrations of apoE protein, inherently rendered lipid load higher in apoE2 and apoE3 treated neurons than in those treated with apoE4 given significantly lower lipidation level of apoE4 (Figures 2A,B). While one can hypothesize equilibration of the lipid load could produce different experimental outcome, such a design could also cause treatment with increased amount of apoE4. The work of Theendakara et al. (2016, 2018) which was based on the expression of apoE4 cDNA in neuroblastoma cell suggests preferential interaction of non-lipidated or lipid-poor apoE with its DNA binding in sequence. Hence, limited lipidation of apoE4 may inherently predispose this isoform to greater degree of interaction with the genomic DNA. Whether and how apoE isoforms differentially engage neuronal transcriptome during aging remains to be experimentally determined but the evidence collected during this study suggests such possibility and this mechanism may play a role in preventing or facilitating age related cognitive decline and neurodegeneration. Thus, our findings support further transcriptomic analyses in aged *APOE*-targeted replacement mice and in human subjects diversified by *APOE* genotype with the objective to identify KEGG signaling pathway and ontological gene categories differentially enriched by various apoE isoforms.

## Conclusion

Our work demonstrates differential regulation of neuronal transcriptome by apoE lipoproteins comprised of various apoE isoforms. This observation evidences a novel apoE-dependent biological mechanism with potential implications for the development and aging of the central nervous system and also contributory to the individual susceptibility to neurodegenerative diseases.

## Data Availability Statement

The original presented in the study are publicly available. This data can be found here: https://www.ncbi.nlm.nih.gov/geo/query/acc.cgi?acc=GSE193162.

## Ethics Statement

The animal study was reviewed and approved by Institutional Animal Care and Use Committees of the New York University Grossman School of Medicine.

## Author Contributions

JD and MS conceived of the project, designed the experiments, and wrote the manuscript. JD and MM-A conducted the research. AH, AT, JP, and PS provided methodological expertise and unique resources. JD, MM-A, AK-J, and MS analyzed the data and designed figures. All authors have read and approved the final version of the manuscript.

## Conflict of Interest

The authors declare that the research was conducted in the absence of any commercial or financial relationships that could be construed as a potential conflict of interest.

## Publisher’s Note

All claims expressed in this article are solely those of the authors and do not necessarily represent those of their affiliated organizations, or those of the publisher, the editors and the reviewers. Any product that may be evaluated in this article, or claim that may be made by its manufacturer, is not guaranteed or endorsed by the publisher.

## Footnotes

1
http://broadinstitute.github.io/picard

2
https://david.ncifcrf.gov/

3
https://www.r-project.org/
