# Supplementary material for: Apolipoprotein E4 Effects a Distinct Transcriptomic Profile and Dendritic Arbor Characteristics in Hippocampal Neurons Cultured in vitro
Source: Front Aging Neurosci. 2022 Apr 29;14:845291. doi: 10.3389/fnagi.2022.845291 (PMC9099260; doi:10.3389/fnagi.2022.845291)
Supplement: Supplementary file 1 [file Data_Sheet_1.pdf]

SUPPLEMENTARY MATERIALS

Supplementary Figure 1

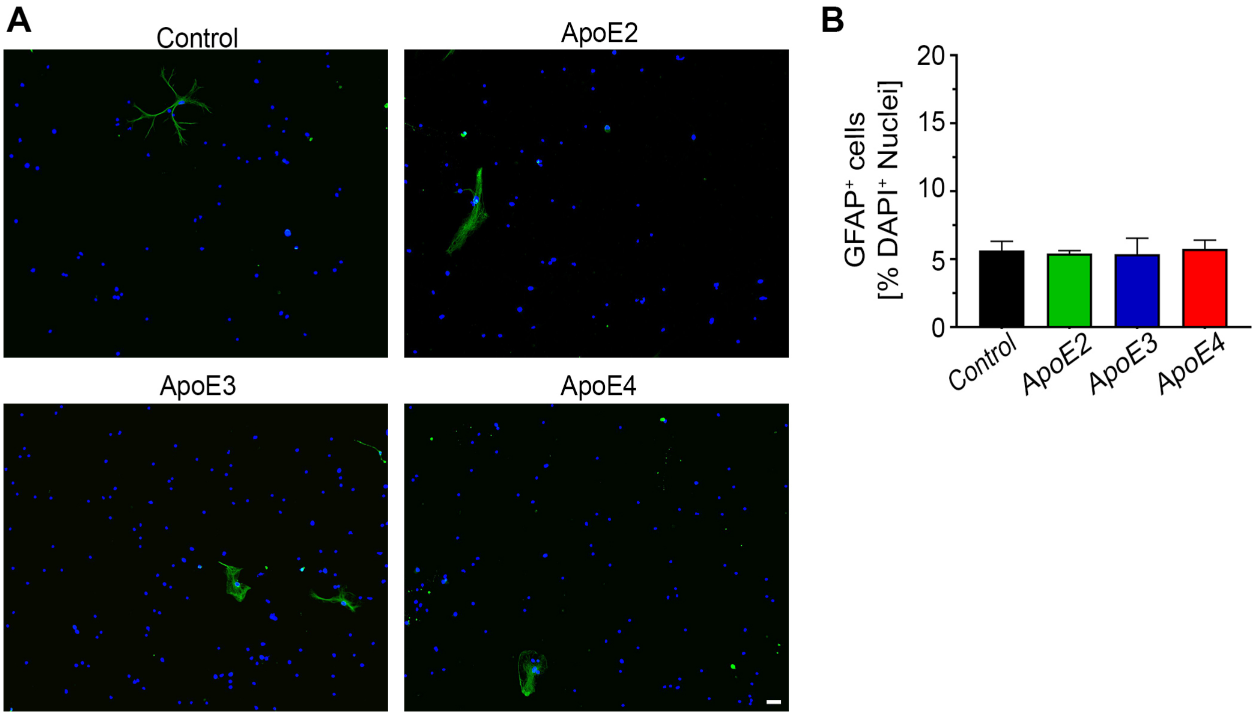

Supplementary Figure 1. Monitoring astrocytic contaminations in dissociated neuronal cultures. **A.** Representative microphotographs of 17 DIV hippocampal primary neuronal cultures grown in the presence of control media (from *Apoe*$^{-/-}$ astrocytes) or indicated apoE lipoproteins, which were stained against GFAP and counterstained with DAPI. **B.** Shown is analysis of the number of GFAP-labeled cells against all cells in the culture, which nuclei were labeled with DAPI. Contamination of dissociated neuronal cultures by native astrocytes is in the range of 5.5% and does not vary across treatment conditions. Values represent mean + SEM from three cultures independently established per each treatment condition. **B** $p = 0.98$ (ANOVA). Scale bar 50 μm in **A**.

Supplementary Table 1. List of transcription factors differentially enriched in primary hippocampal neurons by apoE2, apoE3, or apoE4 lipoproteins. Transcription factors involved in KEGG pathways are highlithed in red. Fold change vs. control neurons grown in the apoE absence. NE - not expressed in the control neurons. *t*-test **p* < 0.05, ***p* < 0.01, ****p* < 0.001, *****p* < 0.0001 vs. control neurons.

Gene	Encoded Protein Name	ApoE2	ApoE3	ApoE4	Function
Ascl2	Achaete-scute family bhlh transcription factor 2	6.6****	7.3****		Neurodevelopment
Cebpb	CCAAT/enhancer-binding protein beta (Kfoury and		2.2*		Synaptic plasticity
Cebpd	CCAAT Enhancer Binding Protein Delta		2.7*		Immune/inflammatory responses
Egr2	E3 SUMO-Protein Transferase ERG2		-2.3*		Myelin formation and maintenance
Elf4	E74-like factor 4 (Kosti et al., 2020)		3.8*	20.9****	Cell cycle
Elk3	ETS Transcription Factor ELK3		2.6*		ERK signaling
Hmga2	High mobility group AT-hook 2		12.2**	3.9*	Cell cycle
Lhx4	LIM/homeobox protein Lhx4		2.5**		Neurodevelopment
Litaf	Lipopolysaccharide-induced tumor necrosis factor-alpha		3.1*		Endosomal/lysosomal pathway
Tcf7	Transcription factor 7		2.9*		Hippo signaling pathway
Tead2	Transcriptional enhancer factor TEF-2		2.7*		Hippo signaling pathway
Tead4	Transcriptional enhancer factor TEF-4		2.5*	2.9**	Hippo signaling pathway
Vdr	Vitamin D3 receptor (Gezen-Ak et al., 2011)	3.0*	6.0***	4.9***	AD pathogenesis
Ets1	E26 avian leukemia oncogene 1, 5' domain			2.6*	Neurodevelopment
Mybl2	Myeloblastosis oncogene-like 2 (Musa et al., 2017)			3.8*	Cell cycle
Elk4	ETS Transcription Factor ELK4			3.5****	ERK pathway and apoptosis
Six4	Sine oculis-related homeobox 4			4.9****	Neurodevelopment
E2f2	E2F transcription factor 2			12.5****	Cell cycle
Runx1	RUNX Family Transcription Factor 1 (Fukui et al., 2018)			2.6*	Reduction of dendritic complexity
Pax3	Paired box 3 (Lin et al., 2016)			NE**	Neurodevelopment
Tead1	TEA domain family member 1			2.3**	Hippo signaling pathway
Gli3	GLI-Kruppel family member GLI3			2.6*	Neurodevelopment
Hoxa3	Homeobox A3			21.1**	Neurodevelopment
E2f7	E2F transcription factor 7			8.0****	Cell cycle
Six3	Sine oculis-related homeobox 3			2.8*	Neurodevelopment
Creb3l3	CAMP Responsive Element Binding Protein 3 Like 3			-2.9*	Endoplasmic reticulum stress response
Klf5	Kruppel Like Factor 5 (Yanagi et al., 2008)			2.1**	Synaptic plasticity
Prrx1	Paired Related Homeobox 1 (Shimozaki et al., 2013)			2.2*	Neuronal cell linage determination/self-
Mafg	MAF BZIP Transcription Factor G (Katsuoka et al., 2003)			2.0****	Glycine signaling

Supplementary Table 2. List of KEGG pathways and individual genes of KEGG pathways, which are differentially enriched by apoE2, apoE3, or apoE4 lipoprotein treatment in primary hippocampal neurons. Fold change vs. control neurons grown in the apoE absence. NE - not expressed in apoE treatment. *t*-test *p < 0.05, **p < 0.01, ***p < 0.001, ****p < 0.0001 vs. control neurons.

Pathway Name	Gene	Encoded Protein Name	*p* value			Fold Change / *p* value		
			ApoE2	ApoE3	ApoE4	ApoE2	ApoE3	ApoE4
PI3K-AKT	*Col4a3*	Collagen, type IV, alpha 3 (Kerrisk et al., 2014, Kurshan and Shen, 2019)	0.0030	0.0012		3.9*		2.8*
	Prlr	Prolactin receptor				2.2*		
	Il7	Interleukin 7				3.7**	5.0**	
	Itga5	Integrin alpha-5 (Kerrisk et al., 2014, Kurshan and Shen, 2019)				2.5*	3.2*	2.6*
	Tnc	Tenascin C (Evers et al., 2002)				2.4*	3.8**	
	Col2a1	Collagen, type II, alpha 1 (Kerrisk et al., 2014, Kurshan and Shen, 2019)				3.4***		
	Ngfr	Tumor necrosis factor receptor superfamily member 16 (Rosch et al., 2005)				3.6**	7.7****	
	Fgf2	Fibroblast growth factor 2 (Woodbury and Ikezu, 2014)				2.2*	3.5**	3.0*
	Il2rb	Interleukin 2 receptor, beta chain (Woodbury and Ikezu, 2014)					7.0**	7.0**
	Flt1	FMS-like tyrosine kinase 1 (Woodbury and Ikezu, 2014)					2.3*	2.6**
	Osmr	Oncostatin-M-specific receptor subunit beta					3.3*	
	Col4a6	Collagen, type IV, alpha 6 (Kerrisk et al., 2014, Kurshan and Shen, 2019)					3.1*	
	Bcl2l11	Bcl-2-like protein 11					2.4****	
	Ccnd1	G1/S-specific cyclin-D1					2.2*	
	Itgav	Integrin alpha-V (Woodbury and Ikezu, 2014) (Kerrisk et al., 2014, Kurshan and Shen, 2019)					2.1*	
	Col6a1	Collagen alpha-1(VI) chain (Kerrisk et al., 2014, Kurshan and Shen, 2019)					2.5**	
	Pik3r5	Phosphoinositide 3-kinase regulatory subunit 5 (Gross and Bassell, 2014)					2.6*	
	Pdgfd	Platelet-derived growth factor D (Woodbury and Ikezu, 2014)					3.1*	
	Spp1	Osteopontin					3.7*	

	Ptger3	Prostaglandin E receptor 3			2.1****		
	Glra1	Glycine receptor subunit alpha-1 (Becker et al., 2006, Horvath et al., 2014)			16.6***	7.9*	
	Prlr	Prolactin receptor			2.2*		
	P2ry2	P2Y purinoceptor 2 (Peterson et al., 2010)			3.0**	5.2***	2.9**
	Tacr1	NK-1 Receptor			2.7**	4.9****	
	Trhr	Thyrotropin-releasing hormone receptor (Daimon et al., 2013)			2.1****	2.2****	
	Bdkrb2	B2 bradykinin receptor			2.1*	3.0**	
	P2ry6	P2Y purinoceptor 6				2.4*	
Neuroactive ligand receptor	*Agtr1b*	Type-1B angiotensin II receptor; Receptor for angiotensin II (Guimond and Gallo-Payet, 2012)	0.0047	0.0230		3.6*	
	Drd2	D(2) dopamine receptor (Linden et al., 2018)				6.8****	
	Glra3	Glycine receptor subunit alpha-3 (McCracken et al., 2017)				3.4***	
	F2rl1	Coagulation factor II (thrombin) receptor-like 1			3.3*	3.9**	
	Ptgfr	Prostaglandin F Receptor				2.2*	
	Sstr5	Somatostatin receptor type 5 (Kailey et al., 2012)			NE*		
	Brs3	Bombesin receptor subtype-3 (Xiao et al., 2017)			NE*		
	Npffr1	Neuropeptide FF receptor 1 (Lin et al., 2017)			-2.1*		
	Glp1r	Glucagon-like peptide 1 receptor (Bliss and Lomo, 1973, Gilman et al., 2003, Abbas et al., 2009)			-3.0*		
	Col4a3	Collagen, type IV, alpha 3			3.9*		2.8*
	Itga5	Integrin alpha-5 (Kerrisk et al., 2014)			2.5*	3.2*	2.6*
	Tnc	Tenascin C			2.4*	3.8**	
	Col2a1	Collagen, type II, alpha 1			3.4***		
	Cd44	CD44 antigen (Skupien et al., 2014, Roszkowska et al., 2016)				2.9*	
ECM-receptor interaction	*Itgav*	Integrin alpha-V	0.0120	0.0072		2.1*	
	Col6a1	Collagen alpha-1(VI) chain				2.5**	
	Col4a6	Collagen, type IV, alpha 6				3.1*	
	Spp1	Osteopontin				3.7*	
	Osmr	Oncostatin-M-specific receptor subunit beta				3.3*	
	Socs3	Suppressor of cytokine signaling 3				3.5**	

	Ctf1	Cardiotrophin-1				3.0*	
	Il21r	Interleukin-21 receptor				4.0*	
	Il19	Interleukin-19				11.0**	
	Pik3r5	Phosphoinositide 3-kinase regulatory subunit 5				2.6*	
	Il13ra1	Interleukin-13 receptor subunit alpha-1				2.4***	
Calcium signaling	*Ptger3*	Prostaglandin E receptor 3	0.0180		2.1****		
	Adcy7	Adenylate cyclase 7 (Sheng et al., 2013)			2.7****		
	Tacr1	Substance-P receptor			2.7**	4.9****	
	Trhr	Thyrotropin-releasing hormone receptor			2.1****	2.2****	
	Bdkrb2	B2 bradykinin receptor			2.1*	3.0**	
Inflamatory regulation of RTP channels	*Adcy7*	Adenylate cyclase 7 (Sheng et al., 2013)	0.0329		2.1****		
	Bdkrb2	B2 bradykinin receptor			2.1*	3.0**	
	P2ry2	P2Y purinoceptor 2 (Peterson et al., 2010)			3.0**	5.2***	2.9**
	Trpa1	Transient receptor potential cation channel, subfamily A, member 1			3.8*		
Cytokine-cytokine receptor interaction	*Il11*	Interlukin 11	0.045	0.0001	4.2*		
	Il12rb1	Interleukin 12 receptor, beta 1			3.3*		
	Il7	Interleukin 7			3.7**	5.0**	
	Ngfr	Tumor necrosis factor receptor superfamily member 16			3.6**	7.7****	
	Prlr	Prolactin receptor			2.2*		
	Bmp2	Bone morphogenetic protein 2				2.7*	
	Ctf1	Cardiotrophin-1				3.0*	
	Clcf1	Cardiotrophin-like cytokine factor 1 (Zou et al., 2009)				4.3*	
	Ccl6	C-C motif chemokine 6			2.5*		
	Il1r1	Interleukin-1 receptor type 1				3.0*	3.0*
	Il13ra1	Interleukin-13 receptor subunit alpha-1				2.4***	
	Il18rap	Interleukin-18 receptor accessory protein				6.2*	5.5*
	Il19	Interleukin-19				11.0**	
	Il2rb	Interleukin 2 receptor, beta chain				7.0**	7.0**

	Il21r	Interleukin-21 receptor					4.0*	
	Lif	LIF Interleukin 6 Family Cytokine					2.4*	
	Osmr	Oncostatin-M-specific receptor subunit beta					3.3*	
	Tnfsf12	Tumor necrosis factor ligand superfamily member 12					3.0*	
	Prlr	Prolactin receptor				2.2*		
	Il7	Interleukin 7				3.7**	5.0**	
	Il11	Interlukin 11					4.2*	
	Lif	LIF Interleukin 6 Family Cytokine					2.4*	
	Il2rb	Interleukin 2 receptor, beta chain					7.0**	7.0**
JAK-STAT	*Ccnd1*	G1/S-specific cyclin-D1	0.0468	0.0001			2.2*	
	Socs3	Suppressor of cytokine signaling 3					3.5**	
	Ctf1	Cardiotrophin-1					3.0*	
	Il21r	Interleukin-21 receptor					4.0*	
	Il19	Interleukin-19					11.0**	
	Pik3r5	Phosphoinositide 3-kinase regulatory subunit 5					2.6*	
	Il13ra1	Interleukin-13 receptor subunit alpha-1					2.4***	
	Bdkrb2	B2 bradykinin receptor				2.1*	3.0**	
	Col4a3	Collagen, type IV, alpha 3				3.9*		2.8*
	Fgf2	Fibroblast growth factor 2				2.2*	3.5**	3.0*
	Wnt5b	Protein Wnt-5b				2.5***	2.5**	
	Agtr1b	Type-1B angiotensin II receptor; Receptor for angiotensin II					3.6*	
	Bmp2	Bone morphogenetic protein 2					2.7*	
Patways in cancer	*Casp8*	Caspase-8		0.0099	0.036		3.2**	
	Col4a6	Collagen, type IV, alpha 6 (Kerrisk et al., 2014, Kurshan and Shen, 2019)					3.1*	
	Ccnd1	G1/S-specific cyclin-D1					2.2*	
	Cdkn2b	Cyclin-dependent kinase 4 inhibitor B (Tawarayama et al., 2019)					2.5*	
	Egln3	Prolyl hydroxylase EGLN3					2.2**	
	Fzd10	Frizzled-10					2.9*	

	Itgav	Integrin alpha-V			2.1*	
	Mmp1a	Interstitial collagenase A			33.7**	15.0*
	Pik3r5	Phosphoinositide 3-kinase regulatory subunit 5			2.6*	
	Pml	Promyelocytic leukemia protein			2.4*	
	Tcf7	Transcription factor 7			2.5*	
	Agtr1b	Type-1B angiotensin II receptor; Receptor for angiotensin II			3.6*	
	Bmp2	Bone morphogenetic protein 2			2.7*	
	Casp8	Caspase-8			3.2**	
	Col4a6	Collagen, type IV, alpha 6			3.1*	
	Fgf2	Fibroblast growth factor 2		2.2*	3.5**	3.0*
	Wnt5b	Protein Wnt-5b		2.5***	2.5**	
	Cbl	Casitas B-lineage lymphoma				2.1***
	E2f2	E2F transcription factor 2				12.5****
	Gli3	GLI-Kruppel family member GLI3				2.6*
	Cdk6	Cyclin-dependent kinase 6				3.8****
	Erbb2	Erb-b2 receptor tyrosine kinase 2				2.5*
	Runx1	Runt-related transcription factor 1				2.6*
	Wnt10b	Wingless-type MMTV integration site family, member 10B				3.0*
TNF signaling	*Bcl3*	B-cell lymphoma 3 protein homolog	0.0001		2.3*	
	Cebpb	CCAAT/enhancer-binding protein beta			2.2*	
	Casp8	Caspase-8			3.2**	
	Lif	Leukemia inhibitory factor			2.4*	2.1*
	Mmp3	Stromelysin-1 (Sedaghat et al., 2012)			4.6*	
	Mapk13	Mitogen-activated protein kinase 13			3.1*	
	Map3k8	Mitogen-activated protein kinase kinase kinase 8			2.7**	
	Nod2	Nucleotide-binding oligomerization domain containing 2			8.3*	9.0*
	Pik3r5	Phosphoinositide 3-kinase regulatory subunit 5			2.6*	
	Sele	E-selectin			3.1*	
	Socs3	Suppressor of cytokine signaling 3			3.5**	

	Cd44	CD44 antigen				2.9*	
	Cav1	Caveolin-1				2.3*	
	Cav2	Caveolin-2				2.0*	
	Ccnd1	G1/S-specific cyclin-D1				2.2*	
	Fgf2	Fibroblast growth factor 2			2.2*	3.5**	3.0*
	Fzd10	Frizzled-10				2.9*	
Proteo-glycans in cancer	*Hbegf*	Proheparin-binding EGF-like growth factor				2.4*	
	Itga5	Integrin alpha-5			2.5*	3.2*	2.6*
	Mapk13	Mitogen-activated protein kinase 13				3.1*	
	Msn	Moesin	0.0002			2.7*	
	Pik3r5	Phosphoinositide 3-kinase regulatory subunit 5				2.6*	
	Rras	Ras-related protein R-Ras				2.2*	
	Wnt5b	Protein Wnt-5b			2.5***	2.5**	
	Fzd10	Frizzled-10				2.9*	
	Tcf7	Transcription factor 7				2.5*	
	Bmp2	Bone morphogenetic protein 2				2.7*	
	Ccnd1	G1/S-specific cyclin-D1				2.2*	
	Wnt5b	Protein Wnt-5b			2.5***	2.5**	
	Ctgf	Connective tissue growth factor			2.8*	4.1*	4.7*
	Id1	Inhibitor of DNA binding 1				5.0**	3.2*
Hippo signaling	*Tead4*	Transcriptional enhancer factor TEF-4 (Lavado et al., 2018)	0.0001	0.0003		2.5*	2.9**
	Tead2	Transcriptional enhancer factor TEF-2 (Lavado et al., 2018)				2.7*	
	Wwtr1	WW domain-containing transcription regulator protein				3.0*	2.1*
	Snai2	Snail family zinc finger 2			3.0*	3.4*	3.2*
	Bmp8a	Bone morphogenetic protein 8A				7.5*	
	Ajuba	Ajuba LIM protein (Lavado et al., 2018)					4.0**
	Wnt10b	Wingless-type MMTV integration site family, member 10B					3.0*
	Mob1a	MOB kinase activator 1A					2.1**

	Tead1	TEA domain family member 1				2.3**	
	Limd1	LIM domains containing 1					2.3**
	Amotl1	Angiomotin-like 1 (Rojek et al., 2019)					2.6**
	Meis1	Homeobox protein Meis1				2.1*	
	Bmp2	Bone morphogenetic protein 2				2.7*	
	Fgf2	Fibroblast growth factor 2			2.2*	3.5**	3.0*
	Fzd10	Frizzled-10				2.9*	
	Id1	Inhibitor of DNA binding 1				5.0**	3.2*
	Id3	DNA-binding protein inhibitor ID-3				6.3****	4.9***
	Lif	Leukemia inhibitory factor				2.4*	2.1*
Pluripotency of stem cells	*Mapk13*	Mitogen-activated protein kinase 13	0.0004	0.0049		3.1*	
	Pik3r5	Phosphoinositide 3-kinase regulatory subunit 5				2.6*	
	Wnt5b	Protein Wnt-5b			2.5***	2.5**	
	Zfhx3	Zinc finger homeobox protein 3				2.8*	4.1***
	Rest	RE1-silencing transcription factor					2.4*
	Wnt10b	Wingless-type MMTV integration site family, member 10B					3.0*
	Zfhx3	Zinc finger homeobox protein 3				2.8*	4.1***
	Cav2	Caveolin-2 (Stern and Mermelstein, 2010)				2.0*	
	Cav1	Caveolin-1 (Stern and Mermelstein, 2010)				2.3*	
	Ccnd1	G1/S-specific cyclin-D1				2.2*	
	Flt1	FMS-like tyrosine kinase 1				2.3*	2.6**
	Itga5	Integrin alpha-5			2.5*	3.2*	2.6*
	Itgav	Integrin alpha-V				2.1*	
Focal adhesion	*Tnc*	Tenascin C	0.0007		2.4*	3.8**	
	Mylk2	Myosin light chain kinase 2, skeletal/cardiac muscle				12.1*	
	Col6a1	Collagen alpha-1(VI) chain				2.5**	
	Pik3r5	Phosphoinositide 3-kinase regulatory subunit 5				2.6*	
	Pdgfd	Platelet-derived growth factor D				3.1*	
	Col4a6	Collagen, type IV, alpha 6				3.1*	
	Spp1	Osteopontin				3.7*	

	Cebpb	CCAAT/enhancer-binding protein beta				2.2*	
	Flt1	FMS-like tyrosine kinase 1				2.3*	2.6**
	Meis1	Homeobox protein Meis1				2.1*	
	Hhex	Hematopoietically-expressed homeobox protein Hhex				12.1*	11.4*
	Hmga2	High mobility group AT-hook 2				12.2**	3.9*
	Hpgd	15-hydroxyprostaglandin dehydrogenase [NAD(+)]			2.5***		
	Il2rb	Interleukin 2 receptor, beta chain				7.0**	7.0**
Transcription misregulation in cancer	*Mmp3*	Stromelysin-1 (Sedaghat et al., 2012)	0.0049	0.0006		4.6*	
	Ngfr	Tumor necrosis factor receptor superfamily member 16			3.6**	7.7****	
	Pml	Promyelocytic leukemia protein				2.4*	
	Aff1	AF4/FMR2 family member 1					3.26****
	Elk4	ETS Transcription Factor ELK4					3.5****
	Hist1h3g	Histone cluster 1, h3g					2.6*
	Il2rb	Interleukin 2 receptor, beta chain				7.0**	7.0**
	Runx1	RUNX Family Transcription Factor 1					2.6*
	Six4	Sine oculis-related homeobox 4					4.9****
	Smad6	MAD homolog 6				3.7*	
	Bmp2	Bone morphogenetic protein 2				2.7*	
	Bmp8a	Bone morphogenetic protein 8A				7.5*	
TGF-beta signaling	*Cdkn2b*	Cyclin-dependent kinase 4 inhibitor B	0.0061			2.5*	
	Id1	Inhibitor of DNA binding 1				5.0**	3.2*
	Id3	DNA-binding protein inhibitor ID-3				6.3****	4.9***
	Ltbp1	Latent-transforming growth factor beta-binding protein 1				3.2**	2.0*
	Arpc1b	Actin-related protein 2/3 complex subunit 1B (Chou and Wang, 2016)				2.8*	
Regulation of actin cytoskeleton	*Itga5*	Integrin alpha-5	0.0086		2.5*	3.2*	2.6*
	Itgav	Integrin alpha-V				2.1*	
	Mylk2	Myosin light chain kinase 2				12.0*	
	Rras	Ras-related protein R-Ras (Iwasawa et al., 2012)				2.2*	

	Pik3r5	Phosphoinositide 3-kinase regulatory subunit 5				2.6*	
	Pdgfd	Platelet-derived growth factor D				3.1*	
	Msn	Moesin				2.7*	
	Bdkrb2	B2 bradykinin receptor			2.1*	3.0**	
	Fgf2	Fibroblast growth factor 2			2.2*	3.5**	3.0*
	Fgd3	FYVE, rhogef and PH domain-containing protein 3				2.4*	
Bacterial invasion of epithelial cells	*Arpc1b*	Actin-related protein 2/3 complex subunit 1B	0.018			2.8*	
	Cav1	Caveolin-1				2.3*	
	Cav2	Caveolin-2				2.0*	
	Itga5	Integrin alpha-5			2.5*	3.2*	2.6*
	Pik3r5	Phosphoinositide 3-kinase regulatory subunit 5				2.6*	
	Rhog	Rho-related GTP-binding protein rhog				2.2*	
Rap1 signaling	*Flt1*	FMS-like tyrosine kinase 1	0.024			2.3*	2.6**
	Angpt2	Angiopoietin-2				2.8*	
	Drd2	D(2) dopamine receptor				6.8****	
	Fgf2	Fibroblast growth factor 2			2.2*	3.5**	3.0*
	Id1	Inhibitor of DNA binding 1				5.0**	3.2*
	Mapk13	Mitogen-activated protein kinase 13				3.1*	
	Ngfr	Tumor necrosis factor receptor superfamily member 16			3.6**	7.7****	
	Pik3r5	Phosphoinositide 3-kinase regulatory subunit 5				2.6*	
	Rras	Ras-related protein R-Ras				2.2*	
MicroRNAs in cancer	*Bmf*	Bcl-2-modifying factor	0.043	0.038		3.0*	4.0***
	Bcl2l11	Bcl-2-like protein 11				2.4****	
	Cd44	CD44 antigen				2.9*	
	Cdc25c	M-phase inducer phosphatase 3				2.3*	
	Ccnd1	G1/S-specific cyclin-D1				2.2*	
	Hmga2	High mobility group AT-hook 2			12.2**	3.9*	
	Itga5	Integrin alpha-5			2.5*	3.2*	2.6*
	Notch3	Neurogenic locus notch homolog protein 3				2.5*	

	Serpinb5	Serine (or cysteine) peptidase inhibitor, clade B, member 5			2.6*	3.4*	
	Tnc	Tenascin C			2.4*	3.8**	
	Vim	Vimentin				2.7*	
	E2f2	E2F transcription factor 2					12.5****
	Cdca5	Cell division cycle associated 5					4.2**
	Cdk6	Cyclin-dependent kinase 6					3.8****
	Erbb2	Erb-b2 receptor tyrosine kinase 2					2.5*
	Igf2bp1	Insulin-like growth factor 2 mrna binding protein 1					55.4****
Toll-like receptor	*Casp8*	Caspase-8	0.047			3.2**	
	Mapk13	Mitogen-activated protein kinase 13				3.1*	
	Map3k8	Mitogen-activated protein kinase kinase kinase 8				2.7**	
	Pik3r5	Phosphoinositide 3-kinase regulatory subunit 5				2.6*	
	Spp1	Osteopontin				3.7*	
	Tlr7	Toll-like receptor 7				4.3***	2.7*
Measles	*Fcgr2b*	Low affinity immunoglobulin gamma Fc region receptor II	0.05			2.5***	
	Ccnd1	G1/S-specific cyclin-D1				2.2*	
	Il2rb	Interleukin 2 receptor, beta chain				7.0**	7.0**
	Msn	Moesin				2.7*	
	Pik3r5	Phosphoinositide 3-kinase regulatory subunit 5				2.6*	
	Tacr1	NK-1 Receptor			2.7**	4.9****	
	Tlr7	Toll-like receptor 7				4.3***	2.7*
HTLV-1 infection	*Bub1b*	Mitotic checkpoint serine/threonine-protein kinase BUB1 beta		0.0041		2.5*	4.1***
	Ets1	E26 avian leukemia oncogene 1, 5' domain					2.6*
	E2f2	E2F transcription factor 2					12.5****
	Elk4	ETS Transcription Factor ELK4					3.5****
	H2-Q6	Histocompatibility 2, Q region locus 6				2.1*	2.4*
	H2-T23	Histocompatibility 2, T region locus 23					2.3*
	H2-T24	Histocompatibility 2, T region locus 24					3.8****
	Il1r1	Interleukin-1 receptor type 1				3.0*	

	Il2rb	Interleukin 2 receptor, beta chain		7.0**	7.0**
	Wnt10b	Wingless-type MMTV integration site family, member 10B			3.0*
Glycerolipid metabolism	*Mboat1*	membrane bound O-acyltransferase domain containing 1 (Tabe et al., 2016)	0.0280		2.9*
	Dgkk	Diacylglycerol kinase kappa (Kim et al., 2010)			2.1*
	Agpat2	1-acylglycerol-3-phosphate O-acyltransferase 2 (lysophosphatidic acid acyltransferase, beta)			2.1*
	Plpp2	phospholipid phosphatase 2 (Kim et al., 2010)			4.2***
Viral carcino-genesis	*Cdk6*	Cyclin-dependent kinase 6	0.0490		3.8****
	H2-Q6	Histocompatibility 2, Q region locus 6		2.1*	2.4*
	H2-T23	Histocompatibility 2, T region locus 23			2.3*
	H2-T24	Histocompatibility 2, T region locus 24			3.8****
	Hist1h2bh	Histone cluster 1, h2bh			2.0*
	Hist1h2bk	Histone cluster 1, h2bk			2.2*
	Hist1h4m	Histone cluster 1, h4m			11.6****
Chronic myeloid leukemia	*Cbl*	Casitas B-lineage lymphoma	0.0490		2.1***
	E2f2	E2F transcription factor 2			12.5****
	Cdk6	Cyclin-dependent kinase 6			3.8****
	Runx1	RUNX Family Transcription Factor 1			2.6*

Supplementary Table 3. List of genes not included in KEGG pathways, which are differentially enriched by apoE2, apoE3, or apoE4 lipoprotein treatment in primary hippocampal neurons. Fold change vs. control neurons grown in the apoE absence. NE - not expressed in the control neurons. *t*-test **p* < 0.05, ***p* < 0.01, ****p* < 0.001, *****p* < 0.0001 vs. control neurons.

Enriched by All Apoliporotein E Isoforms

Ontological Category	Gene	Encoded Protein Name	Fold change / p value		
			ApoE2	ApoE3	ApoE4
Cytoskeleton	*Cdc42bpg*	Serine/threonine-protein kinase MRCK gamma		2.7*	
	Rhoc	Rho-related GTP-binding protein		2.7*	
	Kank2	KN motif and ankyrin repeat domain-containing protein 2		2.6*	
	Rab29	Ras-related protein Rab-7L1		2.5*	
	Plxnb1	Plexin-B1		2.4**	
	Tec	Tyrosine-protein kinase Tec		2.4*	
	Emp2	Epithelial membrane protein 2		2.3*	
	Tes	Testin		2.2*	
	Rhoj	Rho-related GTP-binding protein rhoj (Basu and Lamprecht, 2018)		2.2*	2.1*
	Ninj1	Ninjurin 1	2.1****		
	Gas2l3	Growth arrest-specific 2 like			2.9****
	Wasf2	Wiskott-Aldrich Syndrome Protein (WASP) Family Member 2			2.2*
	Sprr1a	Cornifin-A		4.4*	
	Apbb1ip	Amyloid beta (A4) precursor protein-binding, family B, member 1 interacting protein			3.8*
	Strip2	Striatin-interacting proteins 2		-2.2**	
	Flnc	Filamin C, gamma			2.4*
	Cotl1	Coactosin-like 1	2.3****		
	Fhod3	Formin homology 2 domain containing 3	2.1**	2.4***	
	Krt6a	Keratin, type II cytoskeletal 6A	-8.5*		
	Ecm1	Extracellular matrix protein 1		2.9*	2.1*
	Syne3	Nesprin-3		2.9**	2.6**
	Gan	Giant axonal neuropathy			2.5****
Myelin maintenance	*Gldn*	Gliomedin (Feinberg et al., 2010)	4.1***	6.9****	3.5***
	Egr2	E3 SUMO-Protein Transferase ERG2		-2.3*	

	Prx	Periaxin (Raasakka et al., 2019)			2.8****
	Ascl2	Achaete-scute family bhlh transcription factor 2	6.6****	7.3****	
	Ctf2	Cardiotrophin 2	4.6*		
	Shox2	Short stature homeobox 2 (Scott et al., 2011)			9.6*
	Six4	Sine oculis-related homeobox 4			4.9****
	Bnc2	Basonuclin 2			4.6***
	Id3	DNA-binding protein inhibitor ID-3		6.3****	4.9***
	Gli3	GLI-Kruppel family member GLI3			2.6*
	Notch3	Neurogenic locus notch homolog protein 3		2.5*	
	Pax3	Paired box 3 (Lin et al., 2016)			NE**
	Gpr157	G protein-coupled receptor 157			2.3*
	Zfhx3	Zinc finger homeobox protein 3		2.8*	4.1***
	Ets1	E26 avian leukemia oncogene 1, 5' domain			2.6*
	Papss2	3'-phosphoadenosine 5'-phosphosulfate synthase 2	2.7*	3.7*	
Neurodevelopment	*Kdr*	Vascular endothelial growth factor receptor 2 (Bellon et al., 2010)	-2.3*		
	Igf2bp1	Insulin-like growth factor 2 mrna binding protein 1 (2)			55.4****
	Tll2	Tolloid-like 2	2.1*		
	Hoxa3	Homeobox A3			21.1**
	Zfp568	Zinc finger protein 568			2.8****
	Six3	Sine oculis-related homeobox 3			2.8*
	Mmp21	Matrix metallopeptidase 21			2.6*
	Prdm16	PR domain containing 16		2.2*	2.2*
	Ripply1	Protein ripply1	NE**	NE***	
	Olfml3	Olfactomedin-like protein 3		2.0*	
	Dhh	Desert hedgehog protein		2.5*	
	Phactr4	Phosphatase and actin regulator 4			2.5*
	Dmp1	Dentin matrix protein 1	3.1****		
	Lhx4	LIM/homeobox protein Lhx4		2.5**	
	Serping1	Plasma protease C1 inhibitor (Gorelik et al., 2017)		2.6*	
	Prokr1	Prokineticin receptor 1 (Ruiz-Ferrer et al., 2011)		3.5**	
Immune response	*Ly6g6e*	Lymphocyte antigen 6 complex, locus G6E (Wu et al., 2015)	7.3***	3.2*	
	Cd28	CD28 antigen	3.8*		

	Il11	Interleukin 11	3.6**	4.3*	
	Lime1	Lck-interacting transmembrane adapter 1		7.7*	
	Lypd8	Ly6/PLAUR domain-containing protein 8		7.5***	
	Tmem173	Stimulator of interferon genes protein		4.7**	2.7*
	Tlr7	Toll-like receptor 7 (Hung et al., 2018)		4.3***	2.7*
	Akr1b8	Aldose reductase-related protein 2		2.6*	
	Ptx3	Pentraxin-related protein PTX3 (Rodriguez-Grande et al., 2014)		2.5*	2.3*
	Il33	Interleukin-33		2.1*	
	Clec9a	C-type lectin domain family 9 member A	-4.8**		
	Lif	Leukemia inhibitory factor (Wright et al., 2003)		2.4*	2.1*
	Cebpd	CCAAT Enhancer Binding Protein Delta		2.7*	
	Rbp3	Retinol-binding protein 3 (Komatsu et al., 2005)		10.0*	7.9*
	Lrat	Lecithin retinol acyltransferase (O'Byrne et al., 2005)		5.7*	
	Aldh1a2	Retinal dehydrogenase 2 (Koppaka et al., 2012)		7.1***	
Retinol synthesis/metabolism	*Crabp1*	Cellular retinoic acid binding protein I (Uhrig et al., 2008)	2.7*		2.3*
	Aldh1a3	Retinal dehydrogenase 3 (Koppaka et al., 2012)	11.7**		
	Cyp26b1	Cytochrome P450 26B1(Stoney et al., 2016)	2.9*	6.4****	
	Aox2	Aldehyde oxidase 2	11.7*		
	Cyp26a1	Cytochrome P450 26A1 (Stoney et al., 2016)	2.5*	2.0**	
	Ltbp1	Latent-transforming growth factor beta-binding protein 1		3.2**	2.0*
	Ccdc80	Coiled-coil domain-containing protein 80		3.1*	
	Cdh19	Cadherin 19, type 2		2.9*	2.6
	Scarf2	Scavenger receptor class F, member 2			2.5***
	Plagl2	Pleiomorphic adenoma gene-like 2			2.1***
Cell adhesion	*Lpp*	LIM domain containing preferred translocation partner in lipoma			2.0**
	Troap	Trophinin associated protein			4.7*
	Cdh9	Cadherin-9 (Williams et al., 2011)	-2.1****	-2.3**	
	Rs1	Retinoschisin		-2.6*	
	Cdh23	Cadherin-23	-3.8****	-3.3**	
	Tmem204	Transmembrane protein 204		-4.0*	
	Cldn3	Claudin 3	9.6*		
	Zfp703	Zinc finger protein 703	2.2**	2.1**	2.7**

	Has2	Hyaluronan synthase 2			3.1*
	Ibsp	Bone sialoprotein 2	-2.3*		
	Mcam	Cell surface glycoprotein MUC18		2.4*	
	Ptpn14	Tyrosine-protein phosphatase non-receptor type 14		2.3*	6.4****
	Lrrn4	Leucine-rich repeat neuronal protein 4 (Bando et al., 2013)		-3.0*	
	Fxyd5	FXYD domain-containing ion transport regulator 5 (Tokhtaeva et al., 2016)		2.5*	
	F3	Tissue factor (Chen et al., 2018)		2.8*	
	Cthrc1	Collagen triple helix repeat-containing protein 1		2.4*	
	Loxl4	Loxl4 (Velez et al., 2020)		2.2*	2.7*
	Col13a1	Collagen alpha-1(XIII) chain (Hubert et al., 2009)	-2.5**		
	Col15a1	Collagen alpha-1(XV) chain (Bretaud et al., 2020)	-3.0*		
	Col19a1	Collagen alpha-1(XIX) chain (Su et al., 2010)		-2.3***	
	Mmp1a	Interstitial collagenase A		33.7**	15.0*
	P4ha3	Prolyl 4-hydroxylase subunit alpha-3 (Knapp and Klann, 2002, Salim, 2017)		3.0*	
	Ddr2	Discoidin domain receptor family, member 2			3.1***
	Prkd3	Protein kinase D3			2.4*
	Tgm1	Transglutaminase 1, K polypeptide (Basso et al., 2012)	2.5**	4.5**	4.1*
	Ass1	Argininosuccinate synthase 1 (Heneka et al., 2001)		2.7*	
Oxidative stress	*Cox4i2*	Cytochrome c oxidase subunit IV isoform 2	10.2**		8.9*
	Trim30a	Tripartite motif-containing 30A			2.8*
	Acoxl	Acyl-Coenzyme A oxidase-like Protein kinase D3	NE*		
	Gpx3	Glutathione Peroxidase 3 (Buchser et al., 2012)		3.8**	
	Ncf1	Neutrophil NADPH Oxidase Factor 1 (Brennan et al., 2009)	-2.0*		
	Mdfi	Myod family inhibitor (Chen et al., 2020)		3.2*	
	Sfrp2	Secreted frizzled-related protein 2		3.0*	
	Tpbg	Trophoblast glycoprotein		2.6*	
Wnt signaling	*Wisp1*	WNT1 inducible signaling pathway protein 1 (Maiese, 2014)			2.8*
	Sostdc1	Sclerostin domain containing 1	3.6****		
	Wif1	Wnt inhibitory factor 1	2.3*		
	Nkd1	Protein naked cuticle homolog 1		2.4**	
	Tmem88	Transmembrane protein 88	-2.1*		

	Gpc3	Glypican-3 (Capurro et al., 2014)	-2.9**	-2.9*	
	Mfrp	Membrane frizzled-related protein			2.2*
	Wnt9b	Protein Wnt-9b (Inestrosa and Varela-Nallar, 2014, McLeod and Salinas, 2018)	-2.6*		
	Wnt7a	Protein Wnt-7a (Inestrosa and Varela-Nallar, 2014, McLeod and Salinas, 2018)	-2.0**		
	Gm5415	Predicted gene 5415	-4.2*		
Related to neurological diseases or vision defects	*Vdr*	Vitamin D3 receptor (Gezen-Ak et al., 2011)	3.0*	6.0***	4.9***
	Sh3pxd2a	SH3 and PX domain-containing protein 2A (Saini and Courtneidge, 2018)		2.0**	2.5****
	Dysf	Dysferlin (Galvin et al., 2006)		3.3*	2.9*
	Trib3	Tribbles pseudokinase 3 (Zhang et al., 2019)			2.5*
	Apol9a	Apolipoprotein L 9a (Thekkinghat et al., 2019)			3.3*
	Fbxl7	F-box/LRR-repeat protein 7 (Tosto et al., 2015)	-2.5**		
	Nod2	Nucleotide-binding oligomerization domain containing 2 (28, 29)		8.3*	9.0*
	Mafg	MAF BZIP Transcription Factor G (Katsuoka et al., 2003)			2.0****
	Aoah	Acyloxyacyl hydrolase (Aguiniga et al., 2019)	11.8**	15.5*	14.5*
	Ush1g	Usher syndrome type-1G protein homolog (Weil et al., 2003)	-8.2*		
	Ush2a	Usher syndrome 2A (Weil et al., 2003)		5.8*	7.4**
	Otos	Otospiralin (Delprat et al., 2002)	4.3*	3.2*	
Excitatory signaling	*Slc7a11*	Cystine/Glutamate Transporter (XCR) (Bridges et al., 2012)		2.3*	2.9*
	Spata13	Spermatogenesis-associated protein 13		2.1***	
	Slc30a3	Zinc transporter 3 (Qian and Noebels, 2005, Martel et al., 2010)	-2.4*	-4.1*	
	Pmepa1	Protein TMEPAI (Maag et al., 2015)	2.8****	2.2*	
	Hipk2	Homeodomain interacting protein kinase 2 (Lee et al., 2016, Shang et al., 2018)			4.2****
Survival	*Slc5a3*	Sodium/myo-inositol cotransporter (Miller et al., 1993, Berry et al., 1999, Watanabe et al., 2012, Dai et al., 2016)		2.1*	
	Ngb	Neuroglobin (Sun et al., 2001, Burmester and Hankeln, 2009)		-2.5*	
	Ifi202b	Interferon-activable protein 202 (Xin et al., 2006)	2.3*	3.1*	
	Axl	AXL receptor tyrosine kinase (Axelrod and Pienta, 2014)		2.5*	2.3*
	Prokr2	Prokineticin receptor 2	2.3***	2.0***	
	Otos	Otospiralin	4.3*	3.2*	
	Chp2	Calcineurin B homologous protein 2 (Pang et al., 2002)		2.8**	

	Il1rl1	Interleukin-1 receptor-like 1		2.6*	2.8*
	Cmklr1	Chemerin Chemokine-Like Receptor 1 (Peng et al., 2015)		3.3**	
	Ier3	Radiation-inducible immediate-early gene IEX-1 (Arlt and Schafer, 2011)		2.6*	
	Pde7b	Camp-specific 3’,5’-cyclic phosphodiesterase 7B (Morales-Garcia et al., 2011, Gordon et al., 2016)	-2.1**	-2.7****	-2.1*
	Lgals1	Galectin-1 (Starossom et al., 2012)		3.0*	
	Nxnl2	Nucleoredoxin-like protein 2 (Jaillard et al., 2012)		NE*	
Energy / glucose metabolism	*Gck*	Glucokinase (De Backer et al., 2016, Matschinsky and Wilson, 2019)	-2.2***	-2.1**	
	Medag	Mesenteric estrogen dependent adipogenesis (Zhang et al., 2012)		3.2*	
	Pomc	Pro-opiomelanocortin-alpha (Toda et al., 2017)	2.3*	2.5*	2.8*
Inhibition of neurite growth	*Rgma*	Repulsive guidance molecule A (Isaksen et al., 2020)		2.2*	
	Timp1	Tissue inhibitor of metalloproteinase 1 (Ould-yahoui et al., 2009)	2.9*	4.2**	
	Rhog	Rho-related GTP-binding protein rhog (Franke et al., 2012)		2.2*	
	Hhex	Hematopoietically-expressed homeobox protein Hhex (Simpson et al., 2015)		12.1*	11.4*
Promotion of neurite growth	*Gpr6*	G-protein coupled receptor 6 (Tanaka et al., 2007)		-2.4**	
	Cdh6	Cadherin-6 (Guaiquil et al., 2014, Duan et al., 2018)		2.2*	
	Esm1	Endothelial cell-specific molecule 1		4.6*	
	Kdr	Vascular endothelial growth factor receptor 2 (Luck et al., 2019)	-2.3*		
	Sulf1	Sulfatase 1 (Kalus et al., 2009)	2.0*	2.3*	
	Fpr2	Formyl peptide receptor 2			NE*
Excitatory inhibition	*Gad1-ps*	Glutamate decarboxylase 1, pseudogene (Tao et al., 2018)	3.4*	4.9*	
	Gal	Galanin (Anselmi et al., 2009)	2.9*	4.7**	
	Necab1	N-terminal EF-hand calcium binding protein 1 (Sugita et al., 2002, Lewerenz et al., 2013, Andero et al., 2014, Zhang et al., 2015)	2.1**		
	Gpr39	G protein-coupled receptor 39 (Gilad et al., 2015)			13.2*
	Tac2	Tachykinin-2		3.5*	
	Ano1	Anoctamin-1		4.7**	
	Runx3	Runt related transcription factor 3 (Levanon et al., 2002)	12.0*	30.0**	
Ion channels/transporters	*Atp6v0a4*	V-type proton atpase 116 kda subunit A isoform 4 (Bodzeta et al., 2017, Fassio et al., 2018)		3.5*	
	Clcnkb	Chloride channel protein clc-Kb (Rahmati et al., 2018)		NE*	

	Kcnj8	ATP-sensitive inward rectifier potassium channel 8 (Bajgar et al., 2001, Sun and Feng, 2013)	4.3*	7.6**	
	Gjb6	Connexin 30 (Koulakoff et al., 2008, Pannasch et al., 2014)		5.1*	
	Trpm1	Transient receptor potential cation channel, subfamily M, member 1 (Shen et al., 2012)		3.9*	6.3*
	Kctd4	Potassium Channel Tetramerization Domain Containing 4 (Liu et al., 2013)		-2.2*	
	Kcnmb2	Calcium-activated potassium channel subunit beta-2 (Yu et al., 2018)		-2.3**	
	Trpm3	Transient receptor potential cation channel, subfamily M, member 3 (Zamudio-Bulcock et al., 2011)		-2.3*	
	Slc5a12	Sodium-coupled monocarboxylate transporter 2 (Ganapathy et al., 2008, Riske et al., 2017)	-5.9*		
	Slco1a4	Solute carrier organic anion transporter family member 1A4		3.4*	
	Scn7a	Sodium channel protein		3.1***	
	Piezo2	Piezo-type mechanosensitive ion channel component 2 (Song et al., 2019, Wang and Hamill, 2020)	2.3*		
Endosomes/lysosomes	*Ly75*	Lymphocyte antigen 75		4.5**	3.2*
	Litaf	Lipopolysaccharide-induced tumor necrosis factor-alpha factor homolog (Lee et al., 2011, Lee et al., 2012)		3.1*	
	Ston1	Stonin 1 (Breusegem and Seaman, 2014)			2.4*
	Ifitm1	Interferon-induced transmembrane protein 1		6.5*	5.3*
	Fcgr2b	Low affinity immunoglobulin gamma Fc region receptor II (Kam et al., 2013)		2.5***	
	Asic4	Acid-sensing ion channel 4 (Schwartz et al., 2015)	-2.3**		
	Serpina3g	Serine protease inhibitor A3G		3.0****	2.2*
	Cbl	Casitas B-lineage lymphoma (Yu et al., 2012)			2.1***
	Wipf1	WAS/WASL-interacting protein family member 1 (Franco et al., 2012)		2.5*	2.2*
	Ldlrap1	Low density lipoprotein receptor adaptor protein 1 (Johnson et al., 2014, Lane-Donovan and Herz, 2017)			5.0**
Lipid metabolism	*Lipg*	Endothelial lipase (Yun et al., 2019)		-2.3**	
	Gdpd3	Lysophospholipase D GDPD3 (Lecomte et al., 2005, Wijayatunge et al., 2018)		-3.2*	
	Pla2g2d	Group IID secretory phospholipase A2 (Sun et al., 2010)	-10.4*		
	Liph	Lipase member H (Yung et al., 2015, Roza et al., 2019)		3.5**	
	Gpr132	Probable G-protein coupled receptor 132		13.5**	

	Alox8	Polyunsaturated fatty acid lipoxygenase ALOX8			4.9**
	Alox5	Arachidonate 5-lipoxygenase		3.6*	
	Lcat	Phosphatidylcholine-sterol acyltransferase (Hirsch-Reinshagen et al., 2009)		3.2*	
	Apobr	Apolipoprotein B receptor			2.9*
	Ldlrad4	Low density lipoprotein receptor class A domain containing 4 (Chong et al., 2017)	2.0****		
	Angptl4	Angiopoietin-related protein 4 (Bluher, 2015, Vienberg et al., 2015)		5.0**	4.8*
Cell cycle	*Usp44*	Ubiquitin specific peptidase 44			9.6****
	Prm1	Protamine 1			5.3**
	Cdca5	Cell division cycle associated 5			4.2**
	Kif14	Kinesin family member 14			3.1***
	Aspm	Abnormal spindle-like microcephaly-associated protein homolog			3.0**
	Foxm1	Forkhead box M1			2.6**
	Cdca2	Cell division cycle associated 2			2.5*
	Eme1	Essential meiotic structure-specific endonuclease 1			2.4*
	Nek2	NIMA (never in mitosis gene a)-related expressed kinase 2			2.3***
	Foxn3	Forkhead box N3 (Grassi et al., 2017)			2.1***
	Top2a	Topoisomerase (DNA) II alpha	3.5****		
	Dmc1	Meiotic recombination protein DMC1/LIM15 homolog		-3.2*	
	Meikin	Meiosis-specific kinetochore protein		-5.5*	
	Cdkn3	Cyclin-dependent kinase inhibitor 3	-2.2*		
	Insc	Protein inscuteable homolog	-2.5***		
	Cenpf	Centromere protein F			2.6***
	Rmi2	Recq-mediated			15.7****
	Mybl2	Myeloblastosis oncogene-like 2 (Musa et al., 2017)			3.8*
	Cdkn2b	Cyclin-dependent kinase 4 inhibitor B		2.5*	
	Cdc25c	M-phase inducer phosphatase 3		2.3*	
	Elf4	E74-like factor 4 (Kosti et al., 2020)		3.8*	20.9****
	E2f7	E2F transcription factor 7			8.0****
	Ect2	Ect2 oncogene			3.0*
	Ticrr	TOPBP1-interacting checkpoint and replication regulator			2.8*
	Stil	SCL-interrupting locus protein homolog		2.0*	2.8**

	Tex12	Testis expressed gene 12 (Hamer et al., 2006)	NE**	NE*	
	Cdc6	Cell division cycle 6			5.6****
	E2f2	E2F transcription factor 2 (Szpara et al., 2007, Castillo et al., 2015, Frade and Ovejero-Benito, 2015, Sharma et al., 2017)			12.5****
	Bub1b	Mitotic checkpoint serine/threonine-protein kinase BUB1 beta (Szpara et al., 2007, Frade and Ovejero-Benito, 2015, Sharma et al., 2017)		2.5*	4.1***
	Cdk6	Cyclin-dependent kinase 6 (Sharma et al., 2017)			3.8****
	Espl1	Extra spindle pole bodies 1, separase (Szpara et al., 2007, Frade and Ovejero-Benito, 2015, Sharma et al., 2017)			3.4***

Enriched by apoE2$_{HDL}$ and apoE3$_{HDL}$

Ontological Category	Gene	Encoded Protein Name	ApoE2	ApoE3	ApoE4
Excitatory AMPAR signaling	*Syndig1l*	Synapse differentiation-inducing gene protein 1-like (Kalashnikova et al., 2010)	2.2***	3.6****	
	Gsg1l	Germ cell-specific gene 1-like protein (Gu et al., 2016)	-2.1**	-2.1**	
	Nptx1	Neuronal pentraxin-1 (DeGregorio-Rocasolano et al., 2001, Figueiro-Silva et al., 2015, Schaukowitch et al., 2017)	-2.4*		
	Gpc3	Glypican-3 (Gottschling et al., 2019)	-2.9**	-2.9*	
	Igsf1	Immunoglobulin superfamily member 1 (Jang et al., 2016)	-2.0**		
	Wnt7a	Protein Wnt-7a (McLeod and Salinas, 2018)	-2.1**		
Synaptic plasticity	*C1ql1*	C1q-related factor (Bolliger et al., 2011)		3.1**	
	Pml	Promyelocytic leukemia protein (Korb and Finkbeiner, 2013)		2.4*	
	Akain1	A kinase (PRKA) anchor inhibitor 1 (Zhong et al., 2009)	2.2***		
	Esm1	Endothelial cell-specific molecule 1		4.6*	
	Kdr	Vascular endothelial growth factor receptor 2	-2.3*		
	Klf5	Kruppel Like Factor 5 (Yanagi et al., 2008)		2.1**	
	Spata13	Spermatogenesis-associated protein 13 (Evans et al., 2015)		2.1***	
	Cebpb	CCAAT/enhancer-binding protein beta (Kfoury and Kapatos, 2009)		2.2*	
Neurotransmitter exocytosis	*Syt15*	Synaptotagmin XV (Fukuda, 2003)	2.8*		
	Otof	Otoferlin	-3.3*		
	Stx11	Syntaxin-11 (Johnson and Chapman, 2010, Zhou et al., 2013)		3.6*	
	Synpr	Synaptoporin (Lee et al., 2013, Andre et al., 2018)		-2.4****	

	Sytl4	Synaptotagmin-like protein 4 (Rafi et al., 2019)		2.9*	
	Slc6a2	Sodium-dependent noradrenaline transporter (Torres et al., 2003, Tully and Bolshakov, 2010)		4.6*	
	Rab27b	RAB27B, member RAS oncogene family (Rafi et al., 2019)	2.2***		
Chaperone protein	*Hspb1*	Heat shock protein beta-1 (Srivastava et al., 2012)	2.9*	4.4*	
	Hspb8	Heat shock protein beta-8 (Crippa et al., 2016)		2.9*	
	Hspb3	Heat shock protein 3 (La Padula et al., 2016)	2.8**		
	Fkbp10	Peptidyl-prolyl cis-trans isomerase FKBP10		2.4*	
	Cryaa	Crystallin, alpha A	10.6**		
Cellular growth suppression	*Rasl10a*	Ras-like protein family member 10A (Hermey et al., 2013)	-2.7**	-2.4*	
Tumor suppressor	*Vwa5a*	Von Willebrand factor A domain-containing protein 5A		2.5**	
	Ndrg1	Protein NDRG1 (Okuda et al., 2004, Okuda et al., 2008)		2.2*	

Enriched by apoE3$_{HDL}$ and apoE4$_{HDL}$.

Ontological Category	Gene	Encoded Protein Name	ApoE2	ApoE3	ApoE4
Intracellular trafficking	*Sec16b*	Protein transport protein Sec16B		2.2*	
	Scrg1	Scrapie-responsive protein 1 (Dandoy-Dron et al., 2003)		2.3*	
	Ap1m2	AP-1 complex subunit mu-2		2.4*	
	Klc3	Kinesin light chain 3 (Killian et al., 2012)		2.1**	
	Sec22c	SEC22 homolog C, vesicle trafficking protein (Wang et al., 2018)			2.1****
	Exph5	Exophilin-5 (Ostrowski et al., 2010)		2.1**	
	Sft2d2	SFT2 domain containing 2 (Breusegem and Seaman, 2014)			3.1**
	Ptpn21	Protein tyrosine phosphatase, non-receptor type 21 (Choi et al., 2014)			2.0*
Apoptosis	*Bmf*	Bcl-2-modifying factor (Moran et al., 2013, Akhter et al., 2018)		3.0*	4.0***
	Nradd	Death domain-containing membrane protein NRADD (Wang et al., 2003)		2.9*	
	Casp12	Caspase-12		2.7*	
	Casp8	Caspase-8		3.2**	
	Cideb	Cell death activator CIDE-B		2.2*	
	Zmat3	Zinc finger matrin type 3		2.3*	2.3****

	Adamtsl4	ADAMTS-like protein 4		2.6*	
	Elk4	ETS Transcription Factor ELK4		3.5****	
	Lgals1	Galectin-1		3.0*	
DNA repair	*Gpr87*	G-protein coupled receptor 87 (Zhang et al., 2009)		-16.5*	
	Brip1	BRCA1 interacting protein C-terminal helicase 1 (Greenberg, 2008)			2.7*
	Bard1	BRCA1 associated RING domain 1 (Greenberg, 2008)			2.9**

Enriched by apoE3$_{HDL}$

**Ontological Category**	**Gene**	**Encoded Protein Name**	**ApoE2**	**ApoE3**	**ApoE4**
Lysomal acidification	*Atp6v1c2*	V-type proton atpase subunit C 2		-2.9*	
Lipids synthesis	*Elovl7*	Elongation of very long chain fatty acids protein 7 (Shin et al., 2009)		2.5***	
Development and cognitive impairment	*Fbln2*	Fibulin-2 (Tang et al., 2019)		3.7*	
Calcium storage/sensing	*Casq1*	Calsequestrin-1		4.0*	
	Casr	Extracellular calcium-sensing receptor (Chen et al., 2010)		3.8***	

Enriched by apoE4$_{HDL}$

**Ontological Category**	**Gene**	**Encoded Protein Name**	**ApoE2**	**ApoE3**	**ApoE4**
Histones	*Hist1h2bk*	Histone cluster 1, h2bk (Rachdaoui et al., 2017)			2.2*
	Hist1h2bh	Histone cluster 1, h2bh (Rachdaoui et al., 2017)			2.0*
	Hist1h3g	Histone cluster 1, h3g (Rachdaoui et al., 2017)			2.6*
	Hist1h4m	Histone cluster 1, h4m (Rachdaoui et al., 2017)			11.6****
Increased in aging	*Rest*	RE1-silencing transcription factor (D'Alessandro et al., 2009, Baldelli and Meldolesi, 2015)			2.4*
Fatty acid synthesis in mitochondria	*H2-Ke6*	H2-K region expressed gene 6			4.4*
Stress Response	*Ucn2*	Urocortin 2 (Liu et al., 2015, Zheng et al., 2016)			9.1*
	Creb3l3	CAMP Responsive Element Binding Protein 3 Like 3			-2.9*
Moyamoya disease/ Ischemia	*Rnf213*	Ring finger protein 213 (Sato-Maeda et al., 2016)			2.5*

Nerve regeneration after injury	*Ankrd1*	Ankyrin repeat domain 1 (cardiac muscle) (Obara et al., 2016)	15.9****
Dysregulation synaptic plasticity	*Eif4ebp2*	Eukaryotic translation initiation factor 4E binding protein 2 (Banko et al., 2005)	8.3****

SUPPLEMENTARY REFERENCES

Abbas, T., Faivre, E. and Holscher, C. (2009). Impairment of synaptic plasticity and memory formation in GLP-1 receptor KO mice: Interaction between type 2 diabetes and Alzheimer's disease. *Behav Brain Res.* 205, 265-271. doi: 10.1016/j.bbr.2009.06.035

Aguiniga, L. M., Yang, W., Yaggie, R. E., Schaeffer, A. J., Klumpp, D. J. and Group, M. R. N. S. (2019). Acyloxyacyl hydrolase modulates depressive-like behaviors through aryl hydrocarbon receptor. *Am J Physiol Regul Integr Comp Physiol.* 317, R289-R300. doi: 10.1152/ajpregu.00029.2019

Akhter, R., Saleem, S., Saha, A. and Biswas, S. C. (2018). The pro-apoptotic protein Bmf co-operates with Bim and Puma in neuron death induced by beta-amyloid or NGF deprivation. *Mol Cell Neurosci.* 88, 249-257. doi: 10.1016/j.mcn.2018.02.011

Andero, R., Dias, B. G. and Ressler, K. J. (2014). A role for Tac2, NkB, and Nk3 receptor in normal and dysregulated fear memory consolidation. *Neuron.* 83, 444-454. doi: 10.1016/j.neuron.2014.05.028

Andre, E. A., Forcelli, P. A. and Pak, D. T. (2018). What goes up must come down: homeostatic synaptic plasticity strategies in neurological disease. *Future Neurol.* 13, 13-21. doi: 10.2217/fnl-2017-0028

Anselmi, L., Stella, S. L., Jr., Brecha, N. C. and Sternini, C. (2009). Galanin inhibition of voltage-dependent Ca(2+) influx in rat cultured myenteric neurons is mediated by galanin receptor 1. *J Neurosci Res.* 87, 1107-1114. doi: 10.1002/jnr.21923

Arlt, A. and Schafer, H. (2011). Role of the immediate early response 3 (IER3) gene in cellular stress response, inflammation and tumorigenesis. *Eur J Cell Biol.* 90, 545-552. doi: 10.1016/j.ejcb.2010.10.002

Axelrod, H. and Pienta, K. J. (2014). Axl as a mediator of cellular growth and survival. *Oncotarget.* 5, 8818-8852. doi: 10.18632/oncotarget.2422

Bajgar, R., Seetharaman, S., Kowaltowski, A. J., Garlid, K. D. and Paucek, P. (2001). Identification and properties of a novel intracellular (mitochondrial) ATP-sensitive potassium channel in brain. *J Biol Chem.* 276, 33369-33374. doi: 10.1074/jbc.M103320200

Baldelli, P. and Meldolesi, J. (2015). The Transcription Repressor REST in Adult Neurons: Physiology, Pathology, and Diseases. *eNeuro.* 2 doi: 10.1523/ENEURO.0010-15.2015

Bando, T., Morikawa, Y., Hisaoka, T., Komori, T., Miyajima, A. and Senba, E. (2013). Dynamic expression pattern of leucine-rich repeat neuronal protein 4 in the mouse dorsal root ganglia during development. *Neurosci Lett.* 548, 73-78. doi: 10.1016/j.neulet.2013.05.024

Banko, J. L., Poulin, F., Hou, L., DeMaria, C. T., Sonenberg, N. and Klann, E. (2005). The translation repressor 4E-BP2 is critical for eIF4F complex formation, synaptic plasticity, and memory in the hippocampus. *J Neurosci.* 25, 9581-9590. doi: 10.1523/JNEUROSCI.2423-05.2005

Basso, M., Berlin, J., Xia, L., Sleiman, S. F., Ko, B., Haskew-Layton, R., et al. (2012). Transglutaminase inhibition protects against oxidative stress-induced neuronal death downstream of pathological ERK activation. *J Neurosci.* 32, 6561-6569. doi: 10.1523/JNEUROSCI.3353-11.2012

Basu, S. and Lamprecht, R. (2018). The Role of Actin Cytoskeleton in Dendritic Spines in the Maintenance of Long-Term Memory. *Front Mol Neurosci.* 11, 143. doi: 10.3389/fnmol.2018.00143

Becker, K., Hohoff, C., Schmitt, B., Christen, H. J., Neubauer, B. A., Sandrieser, T., et al. (2006). Identification of the microdeletion breakpoint in a GLRA1null allele of Turkish hyperekplexia patients. *Hum Mutat.* 27, 1061-1062. doi: 10.1002/humu.9455

Bellon, A., Luchino, J., Haigh, K., Rougon, G., Haigh, J., Chauvet, S., et al. (2010). VEGFR2 (KDR/Flk1) signaling mediates axon growth in response to semaphorin 3E in the developing brain. *Neuron.* 66, 205-219. doi: 10.1016/j.neuron.2010.04.006

Berry, G. T., Wang, Z. J., Dreha, S. F., Finucane, B. M. and Zimmerman, R. A. (1999). In vivo brain myo-inositol levels in children with Down syndrome. *J Pediatr.* 135, 94-97. doi: 10.1016/s0022-3476(99)70334-3

Bliss, T. V. and Lomo, T. (1973). Long-lasting potentiation of synaptic transmission in the dentate area of the anaesthetized rabbit following stimulation of the perforant path. *J Physiol.* 232, 331-356. doi: 10.1113/jphysiol.1973.sp010273

Bluher, M. (2015). Distinct roles of angiopoietin-like 4 in the regulation of central and peripheral lipid metabolism? *Mol Metab.* 4, 79-80. doi: 10.1016/j.molmet.2014.12.003

Bodzeta, A., Kahms, M. and Klingauf, J. (2017). The Presynaptic v-ATPase Reversibly Disassembles and Thereby Modulates Exocytosis but Is Not Part of the Fusion Machinery. *Cell Rep.* 20, 1348-1359. doi: 10.1016/j.celrep.2017.07.040

Bolliger, M. F., Martinelli, D. C. and Sudhof, T. C. (2011). The cell-adhesion G protein-coupled receptor BAI3 is a high-affinity receptor for C1q-like proteins. *Proc Natl Acad Sci U S A.* 108, 2534-2539. doi: 10.1073/pnas.1019577108

Brennan, A. M., Suh, S. W., Won, S. J., Narasimhan, P., Kauppinen, T. M., Lee, H., et al. (2009). NADPH oxidase is the primary source of superoxide induced by NMDA receptor activation. *Nat Neurosci.* 12, 857-863. doi: 10.1038/nn.2334

Bretaud, S., Guillon, E., Karppinen, S.-M., Pihlajaniemi, T. and Ruggiero, F. (2020). Collagen XV, a multifaceted multiplexin present across tissues and species. *Matrix Biology Plus.* 6-7, 100023. doi: https://doi.org/10.1016/j.mbplus.2020.100023

Breusegem, S. Y. and Seaman, M. N. J. (2014). Genome-wide RNAi screen reveals a role for multipass membrane proteins in endosome-to-golgi retrieval. *Cell Rep.* 9, 1931-1945. doi: 10.1016/j.celrep.2014.10.053

Bridges, R. J., Natale, N. R. and Patel, S. A. (2012). System xc(-) cystine/glutamate antiporter: an update on molecular pharmacology and roles within the CNS. *Br J Pharmacol.* 165, 20-34. doi: 10.1111/j.1476-5381.2011.01480.x

Buchser, W. J., Smith, R. P., Pardinas, J. R., Haddox, C. L., Hutson, T., Moon, L., et al. (2012). Peripheral nervous system genes expressed in central neurons induce growth on inhibitory substrates. *PLoS One.* 7, e38101. doi: 10.1371/journal.pone.0038101

Burmester, T. and Hankeln, T. (2009). What is the function of neuroglobin? *J Exp Biol.* 212, 1423-1428. doi: 10.1242/jeb.000729

Capurro, M., Martin, T., Shi, W. and Filmus, J. (2014). Glypican-3 binds to Frizzled and plays a direct role in the stimulation of canonical Wnt signaling. *J Cell Sci.* 127, 1565-1575. doi: 10.1242/jcs.140871

Castillo, D. S., Campalans, A., Belluscio, L. M., Carcagno, A. L., Radicella, J. P., Canepa, E. T., et al. (2015). E2F1 and E2F2 induction in response to DNA damage preserves genomic stability in neuronal cells. *Cell Cycle.* 14, 1300-1314. doi: 10.4161/15384101.2014.985031

Chen, C. J., Yang, C. J., Yang, S. F., Huang, M. S. and Liu, Y. P. (2020). The MyoD family inhibitor domain-containing protein enhances the chemoresistance of cancer stem cells in the epithelial state by increasing beta-catenin activity. *Oncogene.* 39, 2377-2390. doi: 10.1038/s41388-019-1152-4

Chen, W., Bergsman, J. B., Wang, X., Gilkey, G., Pierpoint, C. R., Daniel, E. A., et al. (2010). Presynaptic external calcium signaling involves the calcium-sensing receptor in neocortical nerve terminals. *PLoS One.* 5, e8563. doi: 10.1371/journal.pone.0008563

Chen, Y. A., Lu, I. L. and Tsai, J. W. (2018). Contactin-1/F3 Regulates Neuronal Migration and Morphogenesis Through Modulating RhoA Activity. *Front Mol Neurosci.* 11, 422. doi: 10.3389/fnmol.2018.00422

Choi, D. H., Kim, J. H., Seo, J. H., Lee, J., Choi, W. S. and Kim, Y. S. (2014). Matrix metalloproteinase-3 causes dopaminergic neuronal death through Nox1-regenerated oxidative stress. *PLoS One.* 9, e115954. doi: 10.1371/journal.pone.0115954

Chong, J. R., Chai, Y. L., Lee, J. H., Howlett, D., Attems, J., Ballard, C. G., et al. (2017). Increased Transforming Growth Factor beta2 in the Neocortex of Alzheimer's Disease and Dementia with Lewy Bodies is Correlated with Disease Severity and Soluble Abeta42 Load. *J Alzheimers Dis.* 56, 157-166. doi: 10.3233/JAD-160781

Chou, F. S. and Wang, P. S. (2016). The Arp2/3 complex is essential at multiple stages of neural development. *Neurogenesis (Austin).* 3, e1261653. doi: 10.1080/23262133.2016.1261653

Crippa, V., D'Agostino, V. G., Cristofani, R., Rusmini, P., Cicardi, M. E., Messi, E., et al. (2016). Transcriptional induction of the heat shock protein B8 mediates the clearance of misfolded proteins responsible for motor neuron diseases. *Sci Rep.* 6, 22827. doi: 10.1038/srep22827

D'Alessandro, R., Klajn, A. and Meldolesi, J. (2009). Expression of dense-core vesicles and of their exocytosis are governed by the repressive transcription factor NRSF/REST. *Ann N Y Acad Sci.* 1152, 194-200. doi: 10.1111/j.1749-6632.2008.03988.x

Dai, G., Yu, H., Kruse, M., Traynor-Kaplan, A. and Hille, B. (2016). Osmoregulatory inositol transporter SMIT1 modulates electrical activity by adjusting PI(4,5)P2 levels. *Proc Natl Acad Sci U S A.* 113, E3290-3299. doi: 10.1073/pnas.1606348113

Daimon, C. M., Chirdon, P., Maudsley, S. and Martin, B. (2013). The role of Thyrotropin Releasing Hormone in aging and neurodegenerative diseases. *Am J Alzheimers Dis (Columbia).* 1 doi: 10.7726/ajad.2013.1003

Dandoy-Dron, F., Griffond, B., Mishal, Z., Tovey, M. G. and Dron, M. (2003). Scrg1, a novel protein of the CNS is targeted to the large dense-core vesicles in neuronal cells. *Eur J Neurosci.* 18, 2449-2459. doi: 10.1046/j.1460-9568.2003.03009.x

De Backer, I., Hussain, S. S., Bloom, S. R. and Gardiner, J. V. (2016). Insights into the role of neuronal glucokinase. *Am J Physiol Endocrinol Metab.* 311, E42-55. doi: 10.1152/ajpendo.00034.2016

DeGregorio-Rocasolano, N., Gasull, T. and Trullas, R. (2001). Overexpression of neuronal pentraxin 1 is involved in neuronal death evoked by low K(+) in cerebellar granule cells. *J Biol Chem.* 276, 796-803. doi: 10.1074/jbc.M007967200

Delprat, B., Boulanger, A., Wang, J., Beaudoin, V., Guitton, M. J., Venteo, S., et al. (2002). Downregulation of otospiralin, a novel inner ear protein, causes hair cell degeneration and deafness. *J Neurosci.* 22, 1718-1725.

Duan, X., Krishnaswamy, A., Laboulaye, M. A., Liu, J., Peng, Y. R., Yamagata, M., et al. (2018). Cadherin Combinations Recruit Dendrites of Distinct Retinal Neurons to a Shared Interneuronal Scaffold. *Neuron.* 99, 1145-1154 e1146. doi: 10.1016/j.neuron.2018.08.019

Evans, J. C., Robinson, C. M., Shi, M. and Webb, D. J. (2015). The guanine nucleotide exchange factor (GEF) Asef2 promotes dendritic spine formation via Rac activation and spinophilin-dependent targeting. *J Biol Chem.* 290, 10295-10308. doi: 10.1074/jbc.M114.605543

Evers, M. R., Salmen, B., Bukalo, O., Rollenhagen, A., Bosl, M. R., Morellini, F., et al. (2002). Impairment of L-type Ca2+ channel-dependent forms of hippocampal synaptic plasticity in mice deficient in the extracellular matrix glycoprotein tenascin-C. *J Neurosci.* 22, 7177-7194. doi: 20026735

Fassio, A., Esposito, A., Kato, M., Saitsu, H., Mei, D., Marini, C., et al. (2018). De novo mutations of the ATP6V1A gene cause developmental encephalopathy with epilepsy. *Brain.* 141, 1703-1718. doi: 10.1093/brain/awy092

Feinberg, K., Eshed-Eisenbach, Y., Frechter, S., Amor, V., Salomon, D., Sabanay, H., et al. (2010). A glial signal consisting of gliomedin and NrCAM clusters axonal Na+ channels during the formation of nodes of Ranvier. *Neuron.* 65, 490-502. doi: 10.1016/j.neuron.2010.02.004

Figueiro-Silva, J., Gruart, A., Clayton, K. B., Podlesniy, P., Abad, M. A., Gasull, X., et al. (2015). Neuronal pentraxin 1 negatively regulates excitatory synapse density and synaptic plasticity. *J Neurosci.* 35, 5504-5521. doi: 10.1523/JNEUROSCI.2548-14.2015

Frade, J. M. and Ovejero-Benito, M. C. (2015). Neuronal cell cycle: the neuron itself and its circumstances. *Cell Cycle.* 14, 712-720. doi: 10.1080/15384101.2015.1004937

Franco, A., Knafo, S., Banon-Rodriguez, I., Merino-Serrais, P., Fernaud-Espinosa, I., Nieto, M., et al. (2012). WIP is a negative regulator of neuronal maturation and synaptic activity. *Cereb Cortex.* 22, 1191-1202. doi: 10.1093/cercor/bhr199

Franke, K., Otto, W., Johannes, S., Baumgart, J., Nitsch, R. and Schumacher, S. (2012). miR-124-regulated RhoG reduces neuronal process complexity via ELMO/Dock180/Rac1 and Cdc42 signalling. *EMBO J.* 31, 2908-2921. doi: 10.1038/emboj.2012.130

Fukuda, M. (2003). Molecular cloning and characterization of human, rat, and mouse synaptotagmin XV. *Biochem Biophys Res Commun.* 306, 64-71. doi: 10.1016/s0006-291x(03)00911-2

Fukui, H., Runker, A., Fabel, K., Buchholz, F. and Kempermann, G. (2018). Transcription factor Runx1 is pro-neurogenic in adult hippocampal precursor cells. *PLoS One.* 13, e0190789. doi: 10.1371/journal.pone.0190789

Galvin, J. E., Palamand, D., Strider, J., Milone, M. and Pestronk, A. (2006). The muscle protein dysferlin accumulates in the Alzheimer brain. *Acta Neuropathol.* 112, 665-671. doi: 10.1007/s00401-006-0147-8

Ganapathy, V., Thangaraju, M., Gopal, E., Martin, P. M., Itagaki, S., Miyauchi, S., et al. (2008). Sodium-coupled monocarboxylate transporters in normal tissues and in cancer. *AAPS J.* 10, 193-199. doi: 10.1208/s12248-008-9022-y

Gezen-Ak, D., Dursun, E. and Yilmazer, S. (2011). The effects of vitamin D receptor silencing on the expression of LVSCC-A1C and LVSCC-A1D and the release of NGF in cortical neurons. *PLoS One.* 6, e17553. doi: 10.1371/journal.pone.0017553

Gilad, D., Shorer, S., Ketzef, M., Friedman, A., Sekler, I., Aizenman, E., et al. (2015). Homeostatic regulation of KCC2 activity by the zinc receptor mZnR/GPR39 during seizures. *Neurobiol Dis.* 81, 4-13. doi: 10.1016/j.nbd.2014.12.020

Gilman, C. P., Perry, T., Furukawa, K., Grieg, N. H., Egan, J. M. and Mattson, M. P. (2003). Glucagon-like peptide 1 modulates calcium responses to glutamate and membrane depolarization in hippocampal neurons. *J Neurochem.* 87, 1137-1144. doi: 10.1046/j.1471-4159.2003.02073.x

Gordon, R., Neal, M. L., Luo, J., Langley, M. R., Harischandra, D. S., Panicker, N., et al. (2016). Prokineticin-2 upregulation during neuronal injury mediates a compensatory protective response against dopaminergic neuronal degeneration. *Nat Commun.* 7, 12932. doi: 10.1038/ncomms12932

Gorelik, A., Sapir, T., Woodruff, T. M. and Reiner, O. (2017). Serping1/C1 Inhibitor Affects Cortical Development in a Cell Autonomous and Non-cell Autonomous Manner. *Front Cell Neurosci.* 11, 169. doi: 10.3389/fncel.2017.00169

Gottschling, C., Wegrzyn, D., Denecke, B. and Faissner, A. (2019). Elimination of the four extracellular matrix molecules tenascin-C, tenascin-R, brevican and neurocan alters the ratio of excitatory and inhibitory synapses. *Sci Rep.* 9, 13939. doi: 10.1038/s41598-019-50404-9

Grassi, D., Franz, H., Vezzali, R., Bovio, P., Heidrich, S., Dehghanian, F., et al. (2017). Neuronal Activity, TGFbeta-Signaling and Unpredictable Chronic Stress Modulate Transcription of Gadd45 Family Members and DNA Methylation in the Hippocampus. *Cereb Cortex.* 27, 4166-4181. doi: 10.1093/cercor/bhx095

Greenberg, R. A. (2008). Recognition of DNA double strand breaks by the BRCA1 tumor suppressor network. *Chromosoma.* 117, 305-317. doi: 10.1007/s00412-008-0154-8

Gross, C. and Bassell, G. J. (2014). Neuron-specific regulation of class I PI3K catalytic subunits and their dysfunction in brain disorders. *Front Mol Neurosci.* 7, 12. doi: 10.3389/fnmol.2014.00012

Gu, X., Mao, X., Lussier, M. P., Hutchison, M. A., Zhou, L., Hamra, F. K., et al. (2016). GSG1L suppresses AMPA receptor-mediated synaptic transmission and uniquely modulates AMPA receptor kinetics in hippocampal neurons. *Nat Commun.* 7, 10873. doi: 10.1038/ncomms10873

Guaiquil, V. H., Pan, Z., Karagianni, N., Fukuoka, S., Alegre, G. and Rosenblatt, M. I. (2014). VEGF-B selectively regenerates injured peripheral neurons and restores sensory and trophic functions. *Proc Natl Acad Sci U S A.* 111, 17272-17277. doi: 10.1073/pnas.1407227111

Guimond, M. O. and Gallo-Payet, N. (2012). The Angiotensin II Type 2 Receptor in Brain Functions: An Update. *Int J Hypertens.* 2012, 351758. doi: 10.1155/2012/351758

Hamer, G., Gell, K., Kouznetsova, A., Novak, I., Benavente, R. and Hoog, C. (2006). Characterization of a novel meiosis-specific protein within the central element of the synaptonemal complex. *J Cell Sci.* 119, 4025-4032. doi: 10.1242/jcs.03182

Heneka, M. T., Wiesinger, H., Dumitrescu-Ozimek, L., Riederer, P., Feinstein, D. L. and Klockgether, T. (2001). Neuronal and glial coexpression of argininosuccinate synthetase and inducible nitric oxide synthase in Alzheimer disease. *J Neuropathol Exp Neurol.* 60, 906-916. doi: 10.1093/jnen/60.9.906

Hermey, G., Mahlke, C., Gutzmann, J. J., Schreiber, J., Bluthgen, N. and Kuhl, D. (2013). Genome-wide profiling of the activity-dependent hippocampal transcriptome. *PLoS One.* 8, e76903. doi: 10.1371/journal.pone.0076903

Hirsch-Reinshagen, V., Donkin, J., Stukas, S., Chan, J., Wilkinson, A., Fan, J., et al. (2009). LCAT synthesized by primary astrocytes esterifies cholesterol on glia-derived lipoproteins. *J Lipid Res.* 50, 885-893. doi: 10.1194/jlr.M800584-JLR200

Horvath, E., Farkas, K., Herczegfalvi, A., Nagy, N. and Szell, M. (2014). Identification of a novel missense GLRA1 gene mutation in hyperekplexia: a case report. *J Med Case Rep.* 8, 233. doi: 10.1186/1752-1947-8-233

Hubert, T., Grimal, S., Carroll, P. and Fichard-Carroll, A. (2009). Collagens in the developing and diseased nervous system. *Cell Mol Life Sci.* 66, 1223-1238. doi: 10.1007/s00018-008-8561-9

Hung, Y. F., Chen, C. Y., Shih, Y. C., Liu, H. Y., Huang, C. M. and Hsueh, Y. P. (2018). Endosomal TLR3, TLR7, and TLR8 control neuronal morphology through different transcriptional programs. *J Cell Biol.* 217, 2727-2742. doi: 10.1083/jcb.201712113

Inestrosa, N. C. and Varela-Nallar, L. (2014). Wnt signaling in the nervous system and in Alzheimer's disease. *J Mol Cell Biol.* 6, 64-74. doi: 10.1093/jmcb/mjt051

Isaksen, T. J., Fujita, Y. and Yamashita, T. (2020). Repulsive Guidance Molecule A Suppresses Adult Neurogenesis. *Stem Cell Reports.* 14, 677-691. doi: 10.1016/j.stemcr.2020.03.003

Iwasawa, N., Negishi, M. and Oinuma, I. (2012). R-Ras controls axon branching through afadin in cortical neurons. *Mol Biol Cell.* 23, 2793-2804. doi: 10.1091/mbc.E12-02-0103

Jaillard, C., Mouret, A., Niepon, M. L., Clerin, E., Yang, Y., Lee-Rivera, I., et al. (2012). Nxnl2 splicing results in dual functions in neuronal cell survival and maintenance of cell integrity. *Hum Mol Genet.* 21, 2298-2311. doi: 10.1093/hmg/dds050

Jang, S., Oh, D., Lee, Y., Hosy, E., Shin, H., van Riesen, C., et al. (2016). Synaptic adhesion molecule IgSF11 regulates synaptic transmission and plasticity. *Nat Neurosci.* 19, 84-93. doi: 10.1038/nn.4176

Johnson, C. P. and Chapman, E. R. (2010). Otoferlin is a calcium sensor that directly regulates SNARE-mediated membrane fusion. *J Cell Biol.* 191, 187-197. doi: 10.1083/jcb.201002089

Johnson, L. A., Olsen, R. H., Merkens, L. S., DeBarber, A., Steiner, R. D., Sullivan, P. M., et al. (2014). Apolipoprotein E-low density lipoprotein receptor interaction affects spatial memory retention and brain ApoE levels in an isoform-dependent manner. *Neurobiol Dis.* 64, 150-162. doi: 10.1016/j.nbd.2013.12.016

Kailey, B., van de Bunt, M., Cheley, S., Johnson, P. R., MacDonald, P. E., Gloyn, A. L., et al. (2012). SSTR2 is the functionally dominant somatostatin receptor in human pancreatic beta- and alpha-cells. *Am J Physiol Endocrinol Metab.* 303, E1107-1116. doi: 10.1152/ajpendo.00207.2012

Kalashnikova, E., Lorca, R. A., Kaur, I., Barisone, G. A., Li, B., Ishimaru, T., et al. (2010). SynDIG1: an activity-regulated, AMPA-receptor-interacting transmembrane protein that regulates excitatory synapse development. *Neuron.* 65, 80-93. doi: 10.1016/j.neuron.2009.12.021

Kalus, I., Salmen, B., Viebahn, C., von Figura, K., Schmitz, D., D'Hooge, R., et al. (2009). Differential involvement of the extracellular 6-O-endosulfatases Sulf1 and Sulf2 in brain development and neuronal and behavioural plasticity. *J Cell Mol Med.* 13, 4505-4521. doi: 10.1111/j.1582-4934.2008.00558.x

Kam, T. I., Song, S., Gwon, Y., Park, H., Yan, J. J., Im, I., et al. (2013). FcgammaRIIb mediates amyloid-beta neurotoxicity and memory impairment in Alzheimer's disease. *J Clin Invest.* 123, 2791-2802. doi: 10.1172/JCI66827

Katsuoka, F., Motohashi, H., Tamagawa, Y., Kure, S., Igarashi, K., Engel, J. D., et al. (2003). Small Maf compound mutants display central nervous system neuronal degeneration, aberrant transcription, and Bach protein mislocalization coincident with myoclonus and abnormal startle response. *Mol Cell Biol.* 23, 1163-1174. doi: 10.1128/mcb.23.4.1163-1174.2003

Kerrisk, M. E., Cingolani, L. A. and Koleske, A. J. (2014). ECM receptors in neuronal structure, synaptic plasticity, and behavior. *Prog Brain Res.* 214, 101-131. doi: 10.1016/B978-0-444-63486-3.00005-0

Kfoury, N. and Kapatos, G. (2009). Identification of neuronal target genes for CCAAT/enhancer binding proteins. *Mol Cell Neurosci.* 40, 313-327. doi: 10.1016/j.mcn.2008.11.004

Killian, R. L., Flippin, J. D., Herrera, C. M., Almenar-Queralt, A. and Goldstein, L. S. (2012). Kinesin light chain 1 suppression impairs human embryonic stem cell neural differentiation and amyloid precursor protein metabolism. *PLoS One.* 7, e29755. doi: 10.1371/journal.pone.0029755

Kim, K., Yang, J. and Kim, E. (2010). Diacylglycerol kinases in the regulation of dendritic spines. *J Neurochem.* 112, 577-587. doi: 10.1111/j.1471-4159.2009.06499.x

Knapp, L. T. and Klann, E. (2002). Role of reactive oxygen species in hippocampal long-term potentiation: contributory or inhibitory? *J Neurosci Res.* 70, 1-7. doi: 10.1002/jnr.10371

Komatsu, Y., Watakabe, A., Hashikawa, T., Tochitani, S. and Yamamori, T. (2005). Retinol-binding protein gene is highly expressed in higher-order association areas of the primate neocortex. *Cereb Cortex.* 15, 96-108. doi: 10.1093/cercor/bhh112

Koppaka, V., Thompson, D. C., Chen, Y., Ellermann, M., Nicolaou, K. C., Juvonen, R. O., et al. (2012). Aldehyde dehydrogenase inhibitors: a comprehensive review of the pharmacology, mechanism of action, substrate specificity, and clinical application. *Pharmacol Rev.* 64, 520-539. doi: 10.1124/pr.111.005538

Korb, E. and Finkbeiner, S. (2013). PML in the Brain: From Development to Degeneration. *Front Oncol.* 3, 242. doi: 10.3389/fonc.2013.00242

Kosti, A., Du, L., Shivram, H., Qiao, M., Burns, S., Garcia, J. G., et al. (2020). ELF4 Is a Target of miR-124 and Promotes Neuroblastoma Proliferation and Undifferentiated State. *Mol Cancer Res.* 18, 68-78. doi: 10.1158/1541-7786.MCR-19-0187

Koulakoff, A., Ezan, P. and Giaume, C. (2008). Neurons control the expression of connexin 30 and connexin 43 in mouse cortical astrocytes. *Glia.* 56, 1299-1311. doi: 10.1002/glia.20698

Kurshan, P. T. and Shen, K. (2019). Synaptogenic pathways. *Curr Opin Neurobiol.* 57, 156-162. doi: 10.1016/j.conb.2019.03.005

La Padula, V., Staszewski, O., Nestel, S., Busch, H., Boerries, M., Roussa, E., et al. (2016). HSPB3 protein is expressed in motoneurons and induces their survival after lesion-induced degeneration. *Exp Neurol.* 286, 40-49. doi: 10.1016/j.expneurol.2016.08.014

Lane-Donovan, C. and Herz, J. (2017). ApoE, ApoE Receptors, and the Synapse in Alzheimer's Disease. *Trends Endocrinol Metab.* 28, 273-284. doi: 10.1016/j.tem.2016.12.001

Lavado, A., Park, J. Y., Pare, J., Finkelstein, D., Pan, H., Xu, B., et al. (2018). The Hippo Pathway Prevents YAP/TAZ-Driven Hypertranscription and Controls Neural Progenitor Number. *Dev Cell.* 47, 576-591 e578. doi: 10.1016/j.devcel.2018.09.021

Lecomte, M. J., De Gois, S., Guerci, A., Ravassard, P., Faucon Biguet, N., Mallet, J., et al. (2005). Differential expression and regulation of the high-affinity choline transporter CHT1 and choline acetyltransferase in neurons of superior cervical ganglia. *Mol Cell Neurosci.* 28, 303-313. doi: 10.1016/j.mcn.2004.09.014

Lee, K. J., Queenan, B. N., Rozeboom, A. M., Bellmore, R., Lim, S. T., Vicini, S., et al. (2013). Mossy fiber-CA3 synapses mediate homeostatic plasticity in mature hippocampal neurons. *Neuron.* 77, 99-114. doi: 10.1016/j.neuron.2012.10.033

Lee, S., Shang, Y., Redmond, S. A., Urisman, A., Tang, A. A., Li, K. H., et al. (2016). Activation of HIPK2 Promotes ER Stress-Mediated Neurodegeneration in Amyotrophic Lateral Sclerosis. *Neuron.* 91, 41-55. doi: 10.1016/j.neuron.2016.05.021

Lee, S. M., Chin, L. S. and Li, L. (2012). Charcot-Marie-Tooth disease-linked protein SIMPLE functions with the ESCRT machinery in endosomal trafficking. *J Cell Biol.* 199, 799-816. doi: 10.1083/jcb.201204137

Lee, S. M., Olzmann, J. A., Chin, L. S. and Li, L. (2011). Mutations associated with Charcot-Marie-Tooth disease cause SIMPLE protein mislocalization and degradation by the proteasome and aggresome-autophagy pathways. *J Cell Sci.* 124, 3319-3331. doi: 10.1242/jcs.087114

Levanon, D., Bettoun, D., Harris-Cerruti, C., Woolf, E., Negreanu, V., Eilam, R., et al. (2002). The Runx3 transcription factor regulates development and survival of TrkC dorsal root ganglia neurons. *EMBO J.* 21, 3454-3463. doi: 10.1093/emboj/cdf370

Lewerenz, J., Hewett, S. J., Huang, Y., Lambros, M., Gout, P. W., Kalivas, P. W., et al. (2013). The cystine/glutamate antiporter system x(c)(-) in health and disease: from molecular mechanisms to novel therapeutic opportunities. *Antioxid Redox Signal.* 18, 522-555. doi: 10.1089/ars.2011.4391

Lin, J., Wang, C., Yang, C., Fu, S. and Redies, C. (2016). Pax3 and Pax7 interact reciprocally and regulate the expression of cadherin-7 through inducing neuron differentiation in the developing chicken spinal cord. *J Comp Neurol.* 524, 940-962. doi: 10.1002/cne.23885

Lin, Y. T., Yu, Y. L., Hong, W. C., Yeh, T. S., Chen, T. C. and Chen, J. C. (2017). NPFFR2 Activates the HPA Axis and Induces Anxiogenic Effects in Rodents. *Int J Mol Sci.* 18 doi: 10.3390/ijms18081810

Linden, J., James, A. S., McDaniel, C. and Jentsch, J. D. (2018). Dopamine D2 Receptors in Dopaminergic Neurons Modulate Performance in a Reversal Learning Task in Mice. *eNeuro.* 5 doi: 10.1523/ENEURO.0229-17.2018

Liu, C., Liu, X., Song, F., Li, J., Zhang, X. and Yang, J. (2015). The effects of neuropeptide urocortin 2 on the spontaneous discharge and glutamatergic neurotransmission of striatum neurons. *Neuropeptides.* 50, 17-21. doi: 10.1016/j.npep.2015.03.001

Liu, Z., Xiang, Y. and Sun, G. (2013). The KCTD family of proteins: structure, function, disease relevance. *Cell Biosci.* 3, 45. doi: 10.1186/2045-3701-3-45

Luck, R., Urban, S., Karakatsani, A., Harde, E., Sambandan, S., Nicholson, L., et al. (2019). VEGF/VEGFR2 signaling regulates hippocampal axon branching during development. *Elife.* 8 doi: 10.7554/eLife.49818

Maag, J. L., Panja, D., Sporild, I., Patil, S., Kaczorowski, D. C., Bramham, C. R., et al. (2015). Dynamic expression of long noncoding RNAs and repeat elements in synaptic plasticity. *Front Neurosci.* 9, 351. doi: 10.3389/fnins.2015.00351

Maiese, K. (2014). WISP1: Clinical insights for a proliferative and restorative member of the CCN family. *Curr Neurovasc Res.* 11, 378-389. doi: 10.2174/1567202611666140912115107

Martel, G., Hevi, C., Friebely, O., Baybutt, T. and Shumyatsky, G. P. (2010). Zinc transporter 3 is involved in learned fear and extinction, but not in innate fear. *Learn Mem.* 17, 582-590. doi: 10.1101/lm.1962010

Matschinsky, F. M. and Wilson, D. F. (2019). The Central Role of Glucokinase in Glucose Homeostasis: A Perspective 50 Years After Demonstrating the Presence of the Enzyme in Islets of Langerhans. *Front Physiol.* 10, 148. doi: 10.3389/fphys.2019.00148

McCracken, L. M., Lowes, D. C., Salling, M. C., Carreau-Vollmer, C., Odean, N. N., Blednov, Y. A., et al. (2017). Glycine receptor alpha3 and alpha2 subunits mediate tonic and exogenous agonist-induced currents in forebrain. *Proc Natl Acad Sci U S A.* 114, E7179-E7186. doi: 10.1073/pnas.1703839114

McLeod, F. and Salinas, P. C. (2018). Wnt proteins as modulators of synaptic plasticity. *Curr Opin Neurobiol.* 53, 90-95. doi: 10.1016/j.conb.2018.06.003

Miller, B. L., Moats, R. A., Shonk, T., Ernst, T., Woolley, S. and Ross, B. D. (1993). Alzheimer disease: depiction of increased cerebral myo-inositol with proton MR spectroscopy. *Radiology.* 187, 433-437. doi: 10.1148/radiology.187.2.8475286

Morales-Garcia, J. A., Redondo, M., Alonso-Gil, S., Gil, C., Perez, C., Martinez, A., et al. (2011). Phosphodiesterase 7 inhibition preserves dopaminergic neurons in cellular and rodent models of Parkinson disease. *PLoS One.* 6, e17240. doi: 10.1371/journal.pone.0017240

Moran, C., Sanz-Rodriguez, A., Jimenez-Pacheco, A., Martinez-Villareal, J., McKiernan, R. C., Jimenez-Mateos, E. M., et al. (2013). Bmf upregulation through the AMP-activated protein kinase pathway may protect the brain from seizure-induced cell death. *Cell Death Dis.* 4, e606. doi: 10.1038/cddis.2013.136

Musa, J., Aynaud, M. M., Mirabeau, O., Delattre, O. and Grunewald, T. G. (2017). MYBL2 (B-Myb): a central regulator of cell proliferation, cell survival and differentiation involved in tumorigenesis. *Cell Death Dis.* 8, e2895. doi: 10.1038/cddis.2017.244

O'Byrne, S. M., Wongsiriroj, N., Libien, J., Vogel, S., Goldberg, I. J., Baehr, W., et al. (2005). Retinoid absorption and storage is impaired in mice lacking lecithin:retinol acyltransferase (LRAT). *J Biol Chem.* 280, 35647-35657. doi: 10.1074/jbc.M507924200

Obara, Y., Nagasawa, R., Nemoto, W., Pellegrino, M. J., Takahashi, M., Habecker, B. A., et al. (2016). ERK5 induces ankrd1 for catecholamine biosynthesis and homeostasis in adrenal medullary cells. *Cell Signal.* 28, 177-189. doi: 10.1016/j.cellsig.2015.12.016

Okuda, T., Higashi, Y., Kokame, K., Tanaka, C., Kondoh, H. and Miyata, T. (2004). Ndrg1-deficient mice exhibit a progressive demyelinating disorder of peripheral nerves. *Mol Cell Biol.* 24, 3949-3956. doi: 10.1128/mcb.24.9.3949-3956.2004

Okuda, T., Kokame, K. and Miyata, T. (2008). Differential expression patterns of NDRG family proteins in the central nervous system. *J Histochem Cytochem.* 56, 175-182. doi: 10.1369/jhc.7A7323.2007

Ostrowski, M., Carmo, N. B., Krumeich, S., Fanget, I., Raposo, G., Savina, A., et al. (2010). Rab27a and Rab27b control different steps of the exosome secretion pathway. *Nat Cell Biol.* 12, 19-30; sup pp 11-13. doi: 10.1038/ncb2000

Ould-yahoui, A., Tremblay, E., Sbai, O., Ferhat, L., Bernard, A., Charrat, E., et al. (2009). A new role for TIMP-1 in modulating neurite outgrowth and morphology of cortical neurons. *PLoS One.* 4, e8289. doi: 10.1371/journal.pone.0008289

Pang, T., Wakabayashi, S. and Shigekawa, M. (2002). Expression of calcineurin B homologous protein 2 protects serum deprivation-induced cell death by serum-independent activation of Na+/H+ exchanger. *J Biol Chem.* 277, 43771-43777. doi: 10.1074/jbc.M208313200

Pannasch, U., Freche, D., Dallerac, G., Ghezali, G., Escartin, C., Ezan, P., et al. (2014). Connexin 30 sets synaptic strength by controlling astroglial synapse invasion. *Nat Neurosci.* 17, 549-558. doi: 10.1038/nn.3662

Peng, L., Yu, Y., Liu, J., Li, S., He, H., Cheng, N., et al. (2015). The chemerin receptor CMKLR1 is a functional receptor for amyloid-beta peptide. *J Alzheimers Dis.* 43, 227-242. doi: 10.3233/JAD-141227

Peterson, T. S., Camden, J. M., Wang, Y., Seye, C. I., Wood, W. G., Sun, G. Y., et al. (2010). P2Y2 nucleotide receptor-mediated responses in brain cells. *Mol Neurobiol.* 41, 356-366. doi: 10.1007/s12035-010-8115-7

Qian, J. and Noebels, J. L. (2005). Visualization of transmitter release with zinc fluorescence detection at the mouse hippocampal mossy fibre synapse. *J Physiol.* 566, 747-758. doi: 10.1113/jphysiol.2005.089276

Raasakka, A., Linxweiler, H., Brophy, P. J., Sherman, D. L. and Kursula, P. (2019). Direct Binding of the Flexible C-Terminal Segment of Periaxin to beta4 Integrin Suggests a Molecular Basis for CMT4F. *Front Mol Neurosci.* 12, 84. doi: 10.3389/fnmol.2019.00084

Rachdaoui, N., Li, L., Willard, B., Kasumov, T., Previs, S. and Sarkar, D. (2017). Turnover of histones and histone variants in postnatal rat brain: effects of alcohol exposure. *Clin Epigenetics.* 9, 117. doi: 10.1186/s13148-017-0416-5

Rafi, S. K., Fernandez-Jaen, A., Alvarez, S., Nadeau, O. W. and Butler, M. G. (2019). High Functioning Autism with Missense Mutations in Synaptotagmin-Like Protein 4 (SYTL4) and Transmembrane Protein 187 (TMEM187) Genes: SYTL4- Protein Modeling, Protein-Protein Interaction, Expression Profiling and MicroRNA Studies. *Int J Mol Sci.* 20 doi: 10.3390/ijms20133358

Rahmati, N., Hoebeek, F. E., Peter, S. and De Zeeuw, C. I. (2018). Chloride Homeostasis in Neurons With Special Emphasis on the Olivocerebellar System: Differential Roles for Transporters and Channels. *Front Cell Neurosci.* 12, 101. doi: 10.3389/fncel.2018.00101

Riske, L., Thomas, R. K., Baker, G. B. and Dursun, S. M. (2017). Lactate in the brain: an update on its relevance to brain energy, neurons, glia and panic disorder. *Ther Adv Psychopharmacol.* 7, 85-89. doi: 10.1177/2045125316675579

Rodriguez-Grande, B., Swana, M., Nguyen, L., Englezou, P., Maysami, S., Allan, S. M., et al. (2014). The acute-phase protein PTX3 is an essential mediator of glial scar formation and resolution of brain edema after ischemic injury. *J Cereb Blood Flow Metab.* 34, 480-488. doi: 10.1038/jcbfm.2013.224

Rojek, K. O., Krzemien, J., Dolezyczek, H., Boguszewski, P. M., Kaczmarek, L., Konopka, W., et al. (2019). Amot and Yap1 regulate neuronal dendritic tree complexity and locomotor coordination in mice. *PLoS Biol.* 17, e3000253. doi: 10.1371/journal.pbio.3000253

Rosch, H., Schweigreiter, R., Bonhoeffer, T., Barde, Y. A. and Korte, M. (2005). The neurotrophin receptor p75NTR modulates long-term depression and regulates the expression of AMPA receptor subunits in the hippocampus. *Proc Natl Acad Sci U S A.* 102, 7362-7367. doi: 10.1073/pnas.0502460102

Roszkowska, M., Skupien, A., Wojtowicz, T., Konopka, A., Gorlewicz, A., Kisiel, M., et al. (2016). CD44: a novel synaptic cell adhesion molecule regulating structural and functional plasticity of dendritic spines. *Mol Biol Cell.* 27, 4055-4066. doi: 10.1091/mbc.E16-06-0423

Roza, C., Campos-Sandoval, J. A., Gomez-Garcia, M. C., Penalver, A. and Marquez, J. (2019). Lysophosphatidic Acid and Glutamatergic Transmission. *Front Mol Neurosci.* 12, 138. doi: 10.3389/fnmol.2019.00138

Ruiz-Ferrer, M., Torroglosa, A., Nunez-Torres, R., de Agustin, J. C., Antinolo, G. and Borrego, S. (2011). Expression of PROKR1 and PROKR2 in human enteric neural precursor cells and identification of sequence variants suggest a role in HSCR. *PLoS One.* 6, e23475. doi: 10.1371/journal.pone.0023475

Saini, P. and Courtneidge, S. A. (2018). Tks adaptor proteins at a glance. *J Cell Sci.* 131 doi: 10.1242/jcs.203661

Salim, S. (2017). Oxidative Stress and the Central Nervous System. *J Pharmacol Exp Ther.* 360, 201-205. doi: 10.1124/jpet.116.237503

Sato-Maeda, M., Fujimura, M., Kanoke, A., Morita-Fujimura, Y., Niizuma, K. and Tominaga, T. (2016). Transient middle cerebral artery occlusion in mice induces neuronal expression of RNF213, a susceptibility gene for moyamoya disease. *Brain Res.* 1630, 50-55. doi: 10.1016/j.brainres.2015.10.055

Schaukowitch, K., Reese, A. L., Kim, S. K., Kilaru, G., Joo, J. Y., Kavalali, E. T., et al. (2017). An Intrinsic Transcriptional Program Underlying Synaptic Scaling during Activity Suppression. *Cell Rep.* 18, 1512-1526. doi: 10.1016/j.celrep.2017.01.033

Schwartz, V., Friedrich, K., Polleichtner, G. and Grunder, S. (2015). Acid-sensing ion channel (ASIC) 4 predominantly localizes to an early endosome-related organelle upon heterologous expression. *Sci Rep.* 5, 18242. doi: 10.1038/srep18242

Scott, A., Hasegawa, H., Sakurai, K., Yaron, A., Cobb, J. and Wang, F. (2011). Transcription factor short stature homeobox 2 is required for proper development of tropomyosin-related kinase B-expressing mechanosensory neurons. *J Neurosci.* 31, 6741-6749. doi: 10.1523/JNEUROSCI.5883-10.2011

Sedaghat, Y., Mazur, C., Sabripour, M., Hung, G. and Monia, B. P. (2012). Genomic analysis of wig-1 pathways. *PLoS One.* 7, e29429. doi: 10.1371/journal.pone.0029429

Shang, Y., Zhang, J. and Huang, E. J. (2018). HIPK2-Mediated Transcriptional Control of NMDA Receptor Subunit Expression Regulates Neuronal Survival and Cell Death. *J Neurosci.* 38, 4006-4019. doi: 10.1523/JNEUROSCI.3577-17.2018

Sharma, R., Kumar, D., Jha, N. K., Jha, S. K., Ambasta, R. K. and Kumar, P. (2017). Re-expression of cell cycle markers in aged neurons and muscles: Whether cells should divide or die? *Biochim Biophys Acta Mol Basis Dis.* 1863, 324-336. doi: 10.1016/j.bbadis.2016.09.010

Shen, Y., Rampino, M. A., Carroll, R. C. and Nawy, S. (2012). G-protein-mediated inhibition of the Trp channel TRPM1 requires the Gbetagamma dimer. *Proc Natl Acad Sci U S A.* 109, 8752-8757. doi: 10.1073/pnas.1117433109

Sheng, L., Leshchyns'ka, I. and Sytnyk, V. (2013). Cell adhesion and intracellular calcium signaling in neurons. *Cell Commun Signal.* 11, 94. doi: 10.1186/1478-811X-11-94

Shimozaki, K., Clemenson, G. D., Jr. and Gage, F. H. (2013). Paired related homeobox protein 1 is a regulator of stemness in adult neural stem/progenitor cells. *J Neurosci.* 33, 4066-4075. doi: 10.1523/JNEUROSCI.4586-12.2013

Shin, D., Shin, J. Y., McManus, M. T., Ptacek, L. J. and Fu, Y. H. (2009). Dicer ablation in oligodendrocytes provokes neuronal impairment in mice. *Ann Neurol.* 66, 843-857. doi: 10.1002/ana.21927

Simpson, M. T., Venkatesh, I., Callif, B. L., Thiel, L. K., Coley, D. M., Winsor, K. N., et al. (2015). The tumor suppressor HHEX inhibits axon growth when prematurely expressed in developing central nervous system neurons. *Mol Cell Neurosci.* 68, 272-283. doi: 10.1016/j.mcn.2015.08.008

Skupien, A., Konopka, A., Trzaskoma, P., Labus, J., Gorlewicz, A., Swiech, L., et al. (2014). CD44 regulates dendrite morphogenesis through Src tyrosine kinase-dependent positioning of the Golgi. *J Cell Sci.* 127, 5038-5051. doi: 10.1242/jcs.154542

Song, Y., Li, D., Farrelly, O., Miles, L., Li, F., Kim, S. E., et al. (2019). The Mechanosensitive Ion Channel Piezo Inhibits Axon Regeneration. *Neuron.* 102, 373-389 e376. doi: 10.1016/j.neuron.2019.01.050

Srivastava, A. K., Renusch, S. R., Naiman, N. E., Gu, S., Sneh, A., Arnold, W. D., et al. (2012). Mutant HSPB1 overexpression in neurons is sufficient to cause age-related motor neuronopathy in mice. *Neurobiol Dis.* 47, 163-173. doi: 10.1016/j.nbd.2012.03.035

Starossom, S. C., Mascanfroni, I. D., Imitola, J., Cao, L., Raddassi, K., Hernandez, S. F., et al. (2012). Galectin-1 deactivates classically activated microglia and protects from inflammation-induced neurodegeneration. *Immunity.* 37, 249-263. doi: 10.1016/j.immuni.2012.05.023

Stern, C. M. and Mermelstein, P. G. (2010). Caveolin regulation of neuronal intracellular signaling. *Cell Mol Life Sci.* 67, 3785-3795. doi: 10.1007/s00018-010-0447-y

Stoney P. N., Fragoso Y. D., Saeed R. B., Ashton A., Goodman T., et al. (2016). Expression of the retinoic acid catabolic enzyme CYP26B1 in the human brain to maintain signaling homeostasis. *Brain Struct Funct*. 221(6):3315-26. doi: 10.1007/s00429-015-1102-z

Su, J., Gorse, K., Ramirez, F. and Fox, M. A. (2010). Collagen XIX is expressed by interneurons and contributes to the formation of hippocampal synapses. *J Comp Neurol.* 518, 229-253. doi: 10.1002/cne.22228

Sugita, S., Ho, A. and Sudhof, T. C. (2002). NECABs: a family of neuronal Ca(2+)-binding proteins with an unusual domain structure and a restricted expression pattern. *Neuroscience.* 112, 51-63. doi: 10.1016/s0306-4522(02)00063-5

Sun, G. Y., Shelat, P. B., Jensen, M. B., He, Y., Sun, A. Y. and Simonyi, A. (2010). Phospholipases A2 and inflammatory responses in the central nervous system. *Neuromolecular Med.* 12, 133-148. doi: 10.1007/s12017-009-8092-z

Sun, H. S. and Feng, Z. P. (2013). Neuroprotective role of ATP-sensitive potassium channels in cerebral ischemia. *Acta Pharmacol Sin.* 34, 24-32. doi: 10.1038/aps.2012.138

Sun, Y., Jin, K., Mao, X. O., Zhu, Y. and Greenberg, D. A. (2001). Neuroglobin is up-regulated by and protects neurons from hypoxic-ischemic injury. *Proc Natl Acad Sci U S A.* 98, 15306-15311. doi: 10.1073/pnas.251466698

Szpara, M. L., Vranizan, K., Tai, Y. C., Goodman, C. S., Speed, T. P. and Ngai, J. (2007). Analysis of gene expression during neurite outgrowth and regeneration. *BMC Neurosci.* 8, 100. doi: 10.1186/1471-2202-8-100

Tabe, S., Hikiji, H., Ariyoshi, W., Hashidate-Yoshida, T., Shindou, H., Okinaga, T., et al. (2016). Lysophosphatidylethanolamine acyltransferase 1/membrane-bound O-acyltransferase 1 regulates morphology and function of P19C6 cell-derived neurons. *FASEB J.* 30, 2591-2601. doi: 10.1096/fj.201500097R

Tanaka, S., Ishii, K., Kasai, K., Yoon, S. O. and Saeki, Y. (2007). Neural expression of G protein-coupled receptors GPR3, GPR6, and GPR12 up-regulates cyclic AMP levels and promotes neurite outgrowth. *J Biol Chem.* 282, 10506-10515. doi: 10.1074/jbc.M700911200

Tang, C. Z., Yang, J. T., Liu, Q. H., Wang, Y. R. and Wang, W. S. (2019). Up-regulated miR-192-5p expression rescues cognitive impairment and restores neural function in mice with depression via the Fbln2-mediated TGF-beta1 signaling pathway. *FASEB J.* 33, 606-618. doi: 10.1096/fj.201800210RR

Tao, R., Davis, K. N., Li, C., Shin, J. H., Gao, Y., Jaffe, A. E., et al. (2018). GAD1 alternative transcripts and DNA methylation in human prefrontal cortex and hippocampus in brain development, schizophrenia. *Mol Psychiatry.* 23, 1496-1505. doi: 10.1038/mp.2017.105

Tawarayama, H., Feng, Q., Murayama, N., Suzuki, N. and Nakazawa, T. (2019). Cyclin-Dependent Kinase Inhibitor 2b Mediates Excitotoxicity-Induced Death of Retinal Ganglion Cells. *Invest Ophthalmol Vis Sci.* 60, 4479-4488. doi: 10.1167/iovs.19-27396

Thekkinghat, A. A., Yadav, K. K. and Rangarajan, P. N. (2019). Apolipoprotein L9 interacts with LC3/GABARAP and is a microtubule-associated protein with a widespread subcellular distribution. *Biol Open.* 8 doi: 10.1242/bio.045930

Toda, C., Santoro, A., Kim, J. D. and Diano, S. (2017). POMC Neurons: From Birth to Death. *Annu Rev Physiol.* 79, 209-236. doi: 10.1146/annurev-physiol-022516-034110

Tokhtaeva, E., Sun, H., Deiss-Yehiely, N., Wen, Y., Soni, P. N., Gabrielli, N. M., et al. (2016). The O-glycosylated ectodomain of FXYD5 impairs adhesion by disrupting cell-cell trans-dimerization of Na,K-ATPase beta1 subunits. *J Cell Sci.* 129, 2394-2406. doi: 10.1242/jcs.186148

Torres, G. E., Gainetdinov, R. R. and Caron, M. G. (2003). Plasma membrane monoamine transporters: structure, regulation and function. *Nat Rev Neurosci.* 4, 13-25. doi: 10.1038/nrn1008

Tosto, G., Fu, H., Vardarajan, B. N., Lee, J. H., Cheng, R., Reyes-Dumeyer, D., et al. (2015). F-box/LRR-repeat protein 7 is genetically associated with Alzheimer's disease. *Ann Clin Transl Neurol.* 2, 810-820. doi: 10.1002/acn3.223

Tully, K. and Bolshakov, V. Y. (2010). Emotional enhancement of memory: how norepinephrine enables synaptic plasticity. *Mol Brain.* 3, 15. doi: 10.1186/1756-6606-3-15

Uhrig, M., Brechlin, P., Jahn, O., Knyazev, Y., Weninger, A., Busia, L., et al. (2008). Upregulation of CRABP1 in human neuroblastoma cells overproducing the Alzheimer-typical Abeta42 reduces their differentiation potential. *BMC Med.* 6, 38. doi: 10.1186/1741-7015-6-38

Velez, J. I., Lopera, F., Silva, C. T., Villegas, A., Espinosa, L. G., Vidal, O. M., et al. (2020). Familial Alzheimer's Disease and Recessive Modifiers. *Mol Neurobiol.* 57, 1035-1043. doi: 10.1007/s12035-019-01798-0

Vienberg, S. G., Kleinridders, A., Suzuki, R. and Kahn, C. R. (2015). Differential effects of angiopoietin-like 4 in brain and muscle on regulation of lipoprotein lipase activity. *Mol Metab.* 4, 144-150. doi: 10.1016/j.molmet.2014.11.003

Wang, B., Joo, J. H., Mount, R., Teubner, B. J. W., Krenzer, A., Ward, A. L., et al. (2018). The COPII cargo adapter SEC24C is essential for neuronal homeostasis. *J Clin Invest.* 128, 3319-3332. doi: 10.1172/JCI98194

Wang, J. and Hamill, O. P. (2020). Piezo2, a pressure sensitive channel is expressed in select neurons of the mouse brain: a putative mechanism for synchronizing neural networks by transducing intracranial pressure pulses. *bioRxiv.* 2020.2003.2024.006452. doi: 10.1101/2020.03.24.006452

Wang, X., Shao, Z., Zetoune, F. S., Zeidler, M. G., Gowrishankar, K. and Vincenz, C. (2003). NRADD, a novel membrane protein with a death domain involved in mediating apoptosis in response to ER stress. *Cell Death Differ.* 10, 580-591. doi: 10.1038/sj.cdd.4401208

Watanabe, T., Shiino, A. and Akiguchi, I. (2012). Hippocampal metabolites and memory performances in patients with amnestic mild cognitive impairment and Alzheimer's disease. *Neurobiol Learn Mem.* 97, 289-293. doi: 10.1016/j.nlm.2012.01.006

Weil, D., El-Amraoui, A., Masmoudi, S., Mustapha, M., Kikkawa, Y., Laine, S., et al. (2003). Usher syndrome type I G (USH1G) is caused by mutations in the gene encoding SANS, a protein that associates with the USH1C protein, harmonin. *Hum Mol Genet.* 12, 463-471. doi: 10.1093/hmg/ddg051

Wijayatunge, R., Holmstrom, S. R., Foley, S. B., Mgbemena, V. E., Bhargava, V., Perez, G. L., et al. (2018). Deficiency of the Endocytic Protein Hip1 Leads to Decreased Gdpd3 Expression, Low Phosphocholine, and Kypholordosis. *Mol Cell Biol.* 38 doi: 10.1128/MCB.00385-18

Williams, M. E., Wilke, S. A., Daggett, A., Davis, E., Otto, S., Ravi, D., et al. (2011). Cadherin-9 regulates synapse-specific differentiation in the developing hippocampus. *Neuron.* 71, 640-655. doi: 10.1016/j.neuron.2011.06.019

Woodbury, M. E. and Ikezu, T. (2014). Fibroblast growth factor-2 signaling in neurogenesis and neurodegeneration. *J Neuroimmune Pharmacol.* 9, 92-101. doi: 10.1007/s11481-013-9501-5

Wright, L. S., Li, J., Caldwell, M. A., Wallace, K., Johnson, J. A. and Svendsen, C. N. (2003). Gene expression in human neural stem cells: effects of leukemia inhibitory factor. *J Neurochem.* 86, 179-195. doi: 10.1046/j.1471-4159.2003.01826.x

Wu, M., Puddifoot, C. A., Taylor, P. and Joiner, W. J. (2015). Mechanisms of inhibition and potentiation of alpha4beta2 nicotinic acetylcholine receptors by members of the Ly6 protein family. *J Biol Chem.* 290, 24509-24518. doi: 10.1074/jbc.M115.647248

Xiao, C., Pinol, R. A., Carlin, J. L., Li, C., Deng, C., Gavrilova, O., et al. (2017). Bombesin-like receptor 3 (Brs3) expression in glutamatergic, but not GABAergic, neurons is required for regulation of energy metabolism. *Mol Metab.* 6, 1540-1550. doi: 10.1016/j.molmet.2017.08.013

Xin, H., D'Souza, S., Jorgensen, T. N., Vaughan, A. T., Lengyel, P., Kotzin, B. L., et al. (2006). Increased expression of Ifi202, an IFN-activatable gene, in B6.Nba2 lupus susceptible mice inhibits p53-mediated apoptosis. *J Immunol.* 176, 5863-5870. doi: 10.4049/jimmunol.176.10.5863

Yanagi, M., Hashimoto, T., Kitamura, N., Fukutake, M., Komure, O., Nishiguchi, N., et al. (2008). Expression of Kruppel-like factor 5 gene in human brain and association of the gene with the susceptibility to schizophrenia. *Schizophr Res.* 100, 291-301. doi: 10.1016/j.schres.2007.11.042

Yu, M., Guo, L., Li, N., Henzel, K. S., Gu, H., Ran, X., et al. (2018). Overexpression of Kcnmb2 in Dorsal CA1 of Offspring Mice Rescues Hippocampal Dysfunction Caused by a Methyl Donor-Rich Paternal Diet. *Front Cell Neurosci.* 12, 360. doi: 10.3389/fncel.2018.00360

Yu, Y., Sun, Y., He, S., Yan, C., Rui, L., Li, W., et al. (2012). Neuronal Cbl controls biosynthesis of insulin-like peptides in Drosophila melanogaster. *Mol Cell Biol.* 32, 3610-3623. doi: 10.1128/MCB.00592-12

Yun, S. M., Park, J. Y., Seo, S. W. and Song, J. (2019). Association of plasma endothelial lipase levels on cognitive impairment. *BMC Psychiatry.* 19, 187. doi: 10.1186/s12888-019-2174-8

Yung, Y. C., Stoddard, N. C., Mirendil, H. and Chun, J. (2015). Lysophosphatidic Acid signaling in the nervous system. *Neuron.* 85, 669-682. doi: 10.1016/j.neuron.2015.01.009

Zamudio-Bulcock, P. A., Everett, J., Harteneck, C. and Valenzuela, C. F. (2011). Activation of steroid-sensitive TRPM3 channels potentiates glutamatergic transmission at cerebellar Purkinje neurons from developing rats. *J Neurochem.* 119, 474-485. doi: 10.1111/j.1471-4159.2011.07441.x

Zhang, H., Chen, X. and Sairam, M. R. (2012). Novel genes of visceral adiposity: identification of mouse and human mesenteric estrogen-dependent adipose (MEDA)-4 gene and its adipogenic function. *Endocrinology.* 153, 2665-2676. doi: 10.1210/en.2011-2008

Zhang, J., Han, Y., Zhao, Y., Li, Q., Jin, H. and Qin, J. (2019). Inhibition of TRIB3 Protects Against Neurotoxic Injury Induced by Kainic Acid in Rats. *Front Pharmacol.* 10, 585. doi: 10.3389/fphar.2019.00585

Zhang, W., Schmelzeisen, S., Parthier, D., Frings, S. and Mohrlen, F. (2015). Anoctamin Calcium-Activated Chloride Channels May Modulate Inhibitory Transmission in the Cerebellar Cortex. *PLoS One.* 10, e0142160. doi: 10.1371/journal.pone.0142160

Zhang, Y., Qian, Y., Lu, W. and Chen, X. (2009). The G protein-coupled receptor 87 is necessary for p53-dependent cell survival in response to genotoxic stress. *Cancer Res.* 69, 6049-6056. doi: 10.1158/0008-5472.CAN-09-0621

Zheng, Y., Zhang, Y. M. and Ni, X. (2016). Urocortin 2 But Not Urocortin 3 Promotes the Synaptic Formation in Hipppocampal Neurons via Induction of NGF Production by Astrocytes. *Endocrinology.* 157, 1200-1210. doi: 10.1210/en.2015-1812

Zhong, H., Sia, G. M., Sato, T. R., Gray, N. W., Mao, T., Khuchua, Z., et al. (2009). Subcellular dynamics of type II PKA in neurons. *Neuron.* 62, 363-374. doi: 10.1016/j.neuron.2009.03.013

Zhou, P., Bacaj, T., Yang, X., Pang, Z. P. and Sudhof, T. C. (2013). Lipid-anchored SNAREs lacking transmembrane regions fully support membrane fusion during neurotransmitter release. *Neuron.* 80, 470-483. doi: 10.1016/j.neuron.2013.09.010

Zou, X., Bolon, B., Pretorius, J. K., Kurahara, C., McCabe, J., Christiansen, K. A., et al. (2009). Neonatal death in mice lacking cardiotrophin-like cytokine is associated with multifocal neuronal hypoplasia. *Vet Pathol.* 46, 514-519. doi: 10.1354/vp.08-VP-0239-B-BC
